# Mapping the knowledge domains of literature on hepatocellular carcinoma and liver failure: a bibliometric approach

**DOI:** 10.3389/fonc.2025.1529297

**Published:** 2025-04-16

**Authors:** Jun Pu, Yamin Zhao, Siming Zhang, Tianqi Wu, Ruizi Liu, Tianyi Yuan, Songnian He, Qingyu Hao, Haixia Zhu

**Affiliations:** ^1^ Cancer Research Center Nantong, Tumor Hospital Affiliated to Nantong University, Medical School of Nantong University, Nantong, China; ^2^ Department of Cardiology, Nantong Second People's Hospital, Nantong, China; ^3^ Institute of Molecular Biomembrane and Glycobiology, Tohoku Medical and Pharmaceutical University, Sendai, Japan; ^4^ Department of Cardiology, Infectious Disease Hospital of Heilongjiang Province, Harbin, China

**Keywords:** hepatocellular carcinoma, liver failure, bibliometrics, VOSviewer, CiteSpace

## Abstract

**Background:**

Hepatocellular carcinoma (HCC) accounts for 75-85% of primary liver cancers, with its incidence continually rising, posing a threat to socio-economic development. Currently, liver resection is the standard treatment for HCC. However, post-hepatectomy liver failure (PHLF) is a severe and formidable postoperative complication that increases patients’ medical expenses and mortality risk. Additionally, liver failure can occur at any stage of HCC development, severely affecting patients’ quality of life and prognosis.

**Method:**

Using the Web of Science Core Collection, this bibliometric study analyzed English articles and reviews on HCC and liver failure from 2003 to 2023. Bibliometric tools like CiteSpace, VOSviewer, and R-studio were employed for data visualization and analysis, focusing on publication trends, citation metrics, explosive intensity, and collaborative networks. Use the Comparative Toxicogenomics and Genecards databases to screen for genes related to liver failure, and perform enrichment analyses using Gene Ontology, Kyoto Encyclopedia of Genes and Genomes, and PubMed on the identified differentially expressed genes.

**Results:**

The study identified a significant increase in publications on HCC and liver failure, with key contributions from journals such as the World Journal of Gastroenterology and the Journal of Hepatology. The United States, China, and Japan were the leading countries in research output. Prominent authors and institutions, including Kudo Masatoshi and Sun Yat-sen University, were identified. Enrichment analysis showed drug metabolism, oxidative stress, lipid metabolism, and other pathways are closely related to this field. Research hotspots included risk prediction models and novel therapies.

**Conclusion:**

This bibliometric analysis highlights the growing research interest and advancements in HCC and liver failure. Future research should focus on improving risk prediction, developing new therapies, and enhancing international collaboration to address these critical health issues.

## 1 Introduction

Hepatocellular carcinoma (HCC) represents the predominant form of primary malignant neoplasm of the liver, constituting approximately 75-85% of all liver cancer diagnoses (1). The disease is characterized by a high prevalence and a significant mortality rate (2). Currently, notable treatment modalities for HCC encompass surgical resection, liver transplantation, local ablation, radiation therapy, and systemic therapies. However, due to the limitations imposed by liver failure, many patients cannot tolerate these treatments (3). Furthermore, common surgical treatments may lead to postoperative complications such as post-hepatectomy liver failure (PHLF), resulting in prolonged hospitalization, poor prognosis, and increased mortality risk (4). Reports indicate that the success of Associating Liver Partition and Portal Vein Ligation for Staged Hepatectomy (ALPPS) is critically dependent on the liver’s regenerative capacity. Insufficient liver regeneration to restore hepatic function may result in liver failure, potentially leading to fatal outcomes (5, 6). In summary, the occurrence of liver failure profoundly influences the therapeutic approaches and the overall prognosis for patients afflicted with HCC. Therefore, early identification of risk factors for liver failure, timely diagnosis, and treatment are crucial to prevent liver failure, reduce HCC risk, and improve patient outcomes. It is imperative to diligently circumvent and adeptly handle post-surgical complications, while concurrently devising efficacious measures for preemption and intervention.

Bibliometrics is the quantitative analysis of scientific literature, focusing on metrics such as citation counts, publication counts, and co-authorship patterns. This field helps understand research trends, evaluate scientific impact, and inform policy decisions (7). As a critical tool in research assessment, bibliometrics aids in mapping the structure and dynamics of scientific knowledge. Hepatic failure is a severe complication following HCC surgery and affects the entire process of hepatocarcinogenesis. Moreover, the current treatment and detection methods for HCC and liver failure have obvious deficiencies in tolerance and specificity. Research in this area will contribute to preventing this condition, improving patient prognosis, and expanding treatment options. It is noteworthy that previous studies have predominantly focused on clinical aspects of HCC or liver failure in isolation, this is the first bibliometric study to systematically map the knowledge domains bridging HCC and liver failure over 20 years (2003–2023), revealing their evolving synergies in research trends, collaborative networks, and therapeutic advancements. By synergistically employing multiple visualization tools (CiteSpace, VOSviewer, R-Studio, and Scimago), we not only quantify publication metrics but also decode interdisciplinary linkages, emerging hotspots, such as immunotherapy and precision medicine, as well as global collaboration patterns, offering a multidimensional perspective. In addition to descriptive statistics, we discussed the signaling pathways of gene enrichment related to liver failure in liver cancer, further clarified its tissue metabolism and pathogenesis, and identified areas of insufficient exploration such as drug-induced liver injury in targeted therapy, which provided operational guidance for future research and clinical practice. Through burst detection and timeline clustering, we delineate three distinct research phases: risk factor exploration (pre-2011), therapeutic innovation (2012–2017), and personalized medicine (post-2018), highlighting paradigm shifts in the field.

## 2 Methods

### 2.1 Data sources and search strategies

**Figure 1:**
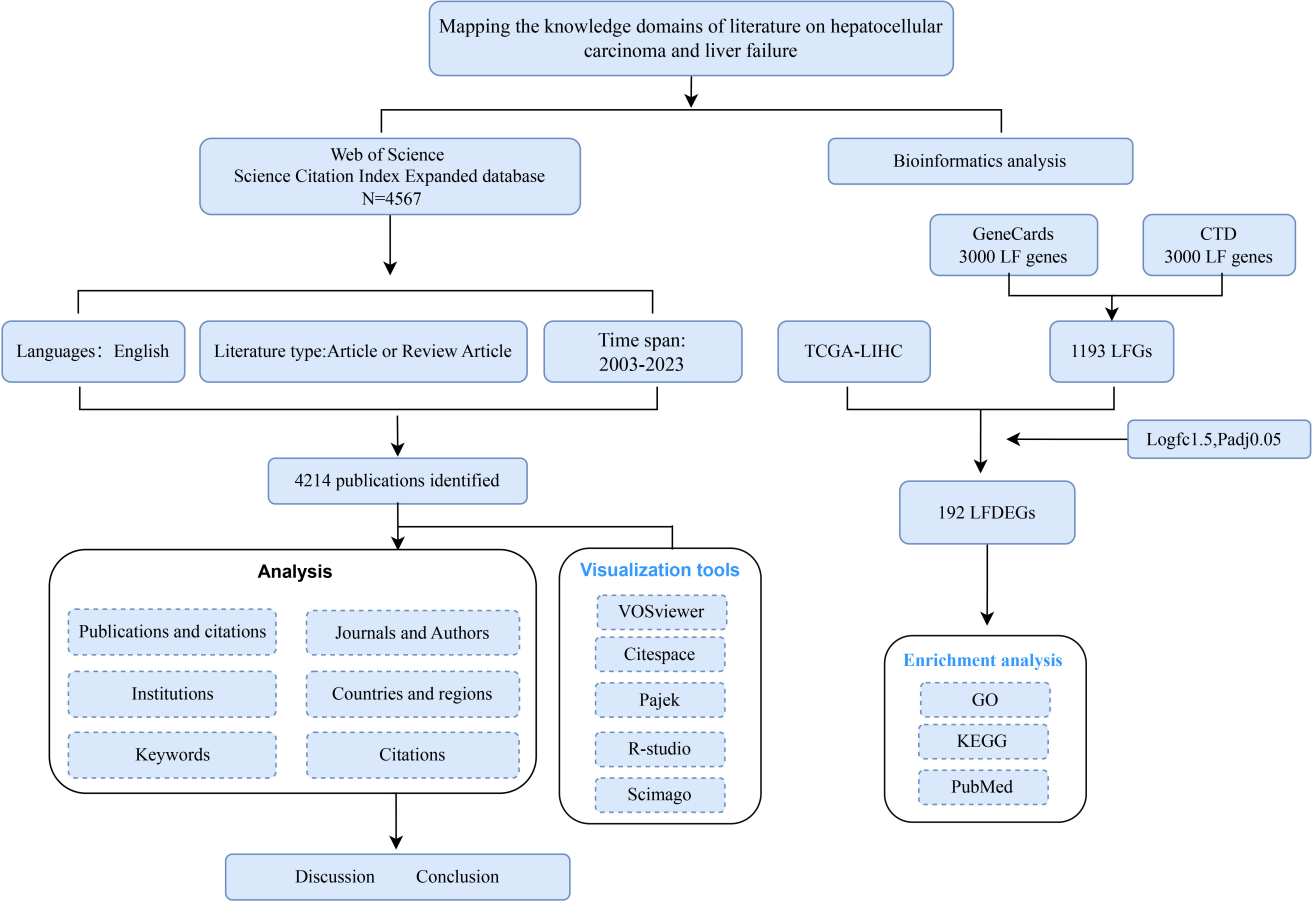
Flowchart of hepatocellular carcinoma and liver failure.

The Web of Science (WoS) is recognized as the most extensively utilized database by researchers for obtaining global research data and is considered an authoritative source for literature in this domain (7). In this study, we extracted literature and data from the WoSCC Science Citation Index Expanded database and conducted subsequent analyses. All searches and data downloads were completed within a single day to minimize the risk of discrepancies arising from database updates. We employed both controlled and free terms related to HCC and liver failure (SDC, **Table 1**). The specific literature analysis process is shown in **Figure 1**. The articles included in the analysis were published between January 1, 2003, and December 31, 2023. Only original research articles and review papers published in English were selected for inclusion. The exported data comprises “full records and citations” in “plain text” format, including both complete records and cited references.

**Table 1:** Top 10 journals in terms of citations.

Ranking	Journal	Record Count	Citations	IF	JCR	H-index	Country
1	Journal Of Hepatology	92	11347	26.8	Q1	216	Netherlands
2	Hepatology	81	9829	12.9	Q1	326	United States
3	Annals Of Surgery	33	5340	7.5	Q1	284	United States
4	World Journal of Gastroenterology	124	4183	4.3	Q2	129	China
5	Lancet	6	3872	98.4	Q1	700	United Kingdom
6	Liver Transplantation	60	3739	4.7	Q1	137	United States
7	Lancet Oncology	8	3223	41.6	Q1	274	United Kingdom
8	Liver International	77	2808	6	Q1	98	United States
9	Gastroenterology	18	2739	25.7	Q1	368	United States
10	Gut	15	2575	23	Q1	262	United Kingdom

### 2.2 Software for bibliometric analysis and visualization analysis

In this research, the suite of data analysis and visualization tools comprises Microsoft Office Excel 2021, CiteSpace 6.3.R3 (8), VOSviewer 1.6.20 (9), Pajek, Scimago, and R-Studio. Specifically, Excel 2021 was utilized for the compilation of trend statistics, the structuring of data, and the generation of pertinent tables. The function of VOSviewer is based on its embedded clustering algorithm (9, 10). It is a potent tool for co-authorship analysis and cooccurrence analysis (11). This paper utilizes VOSviewer to illustrate the collaborative networks among journals, authors, institutions, and countries using three visualization methods: network visualization, overlay visualization, and density visualization. Each node in the figure corresponds to a specific journal, author, institution, or country. In the network visualization, each element is represented by a circle and label, with the size of the element determined by factors such as degree, link strength, and citation count. The color of each element represents its cluster, with different clusters indicated by distinct colors. For example, thematic co-occurrence can reveal the structural distribution of research hotspots, author collaborations can uncover small research groups, and author coupling networks can highlight similarities and differences in scholars’ approaches to research topics. In the overlay visualization, color changes reflect the spatial mapping of nodes based on their average publication year. In the density visualization, density is determined by the number of elements in the surrounding area and their significance. The density view enables a quick assessment of key fields and the knowledge or research density within a particular area. CiteSpace is also a commonly used literature visualization software (8), which was employed to analyze countries, regions, co-cited references, and keywords, and to create visual maps. Using Scimago, a cooperation network relationship among the top thirty countries in this field was drawn based on national citation data. Each node represents a country, the size of the node indicates the number of citations, and the thickness of the connecting line represents the degree of closeness of cooperation and communication. R-Studio is an excellent software that integrates data operation, statistics, and visualization functions. It was used to plot author publication trends and keyword trends in the article. Pajek is used for image layout adjustment.

### 2.3 Bioinformatics analysis

The RNA-seq data for HCC were obtained from The Cancer Genome Atlas (TCGA-LIHC) database, comprising 50 normal liver tissues and 374 tumor tissues. Principal component analysis (PCA) was performed to assess the overall distribution of the selected count data. To identify genes associated with liver failure, the top 3,000 genes ranked by relevance score from GeneCards (12, https://www.genecards.org/) and the top 3,000 genes ranked by Inference Score from the Comparative Toxicogenomics Database (CTD) (13, http://ctdbase.org/) were intersected to construct a liver failure gene set. Differentially expressed genes (DEGs) between normal and tumor tissues were identified using DESeq2, with selection criteria of Log2 fold change |logFC| > 1.5 and an adjusted p-value (padj) < 0.05. The identified liver failure-associated DEGs were subsequently analyzed through Gene Ontology (GO), Kyoto Encyclopedia of Genes and Genomes (KEGG) pathway, and PubMed enrichment analyses. All statistical analyses were performed using R tools.

## 3 Results

### 3.1 Publication and citation

**Figure 2:**
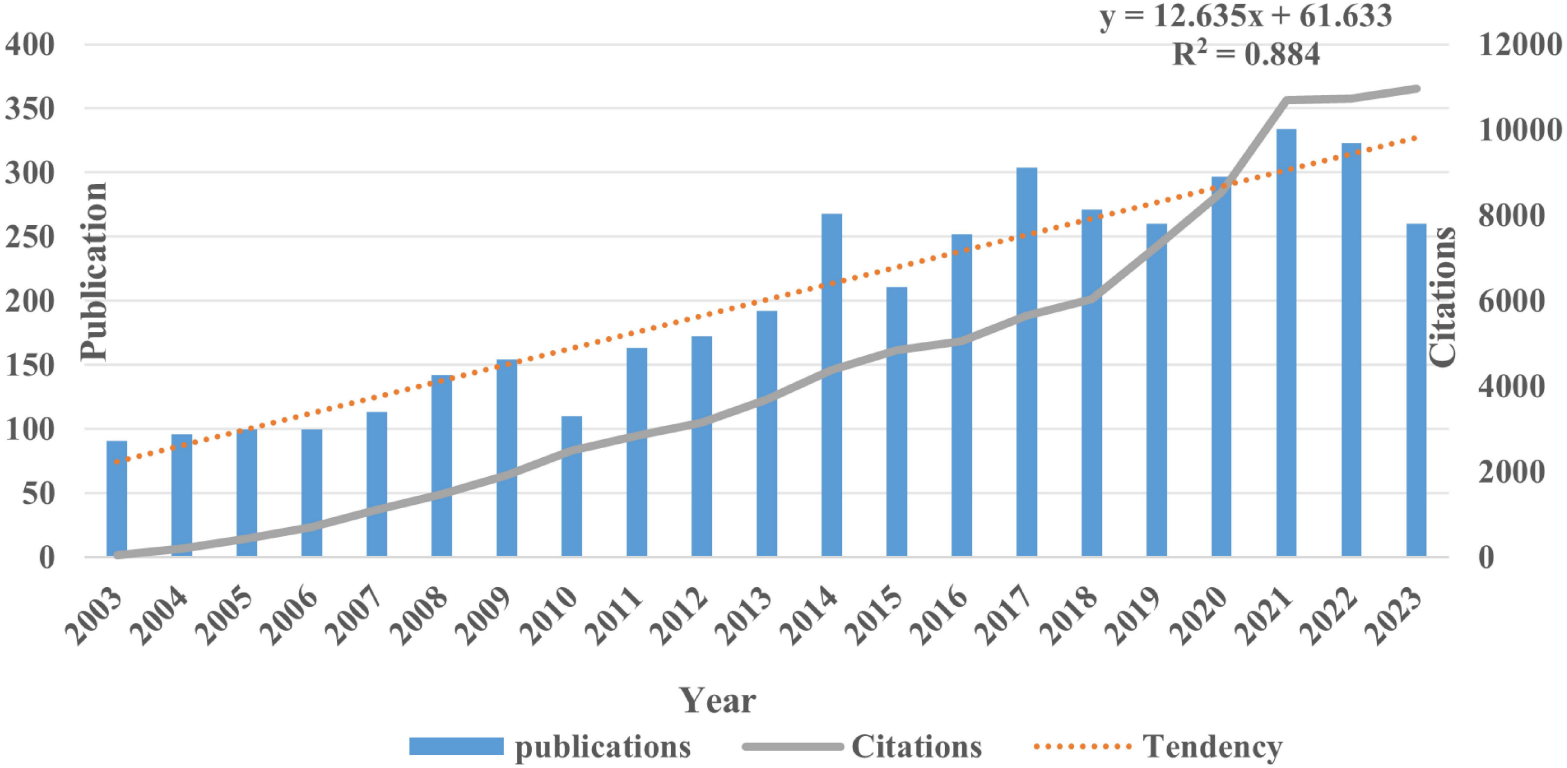
Publication and citations of hepatocellular carcinoma and liver failure. The citation trend line of the publication volume shows an exponential growth pattern. The line graph illustrates a year-on-year increase in the number of citations.

From January 2003 to December 2023, a total of 4214 publications on the theme of hepatocellular carcinoma and liver failure were retrieved, over a period of 20 years. To enhance comprehension of the evolution pattern of hepatocellular carcinoma and liver failure, we have created a graph illustrating the yearly quantity of publications and citations in this domain (**Figure 2**). The body of research on liver failure due to HCC has demonstrated a notable annual increase in publication volume. In recent years, the quantity of publications has remained high, indicative of growing interest and activity among academics in this topic. Regarding citation frequency, the number of citations varies from year to year, even as the total volume of publications rises. For instance, although 2022 saw a higher number of publications overall, the average citation frequency was only three times, suggesting that the literature from that year requires more time to establish its scholarly impact.

### 3.2 Journal analysis

**Figure 3:**
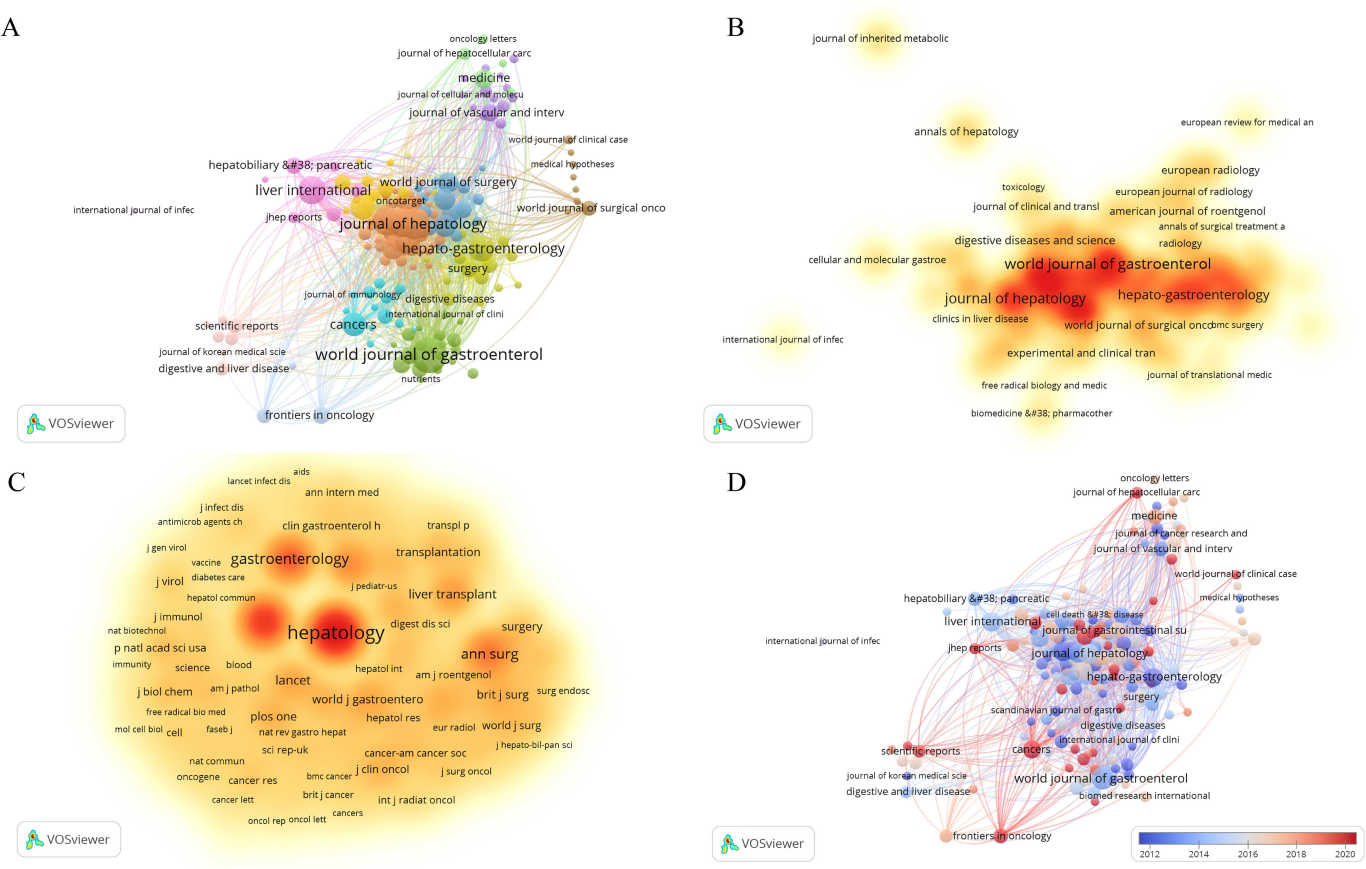
Network visualization of hepatocellular carcinoma and liver failure. **(A)** Collaborative network map of journals with ≥9 publications. Each node represents a journal, with the node’s size indicating the number of publications, and the connections between nodes representing collaborative relationships. **(B)** Map of core journals in hepatocellular carcinoma and liver failure. **(C)** Map of co-cited journals in hepatocellular carcinoma and liver failure. The nodes with darker colors represent greater importance in the field. **(D)** Map of emerging journals in hepatocellular carcinoma and liver failure. The color variation reflects the spatial mapping of nodes based on the average publication year of co-cited journals.

To choose the analysis type and counting method (Citation-Sources), we imported the data into the VOSviewer software. After doing so, articles from the top 182 journals contributing to the study of HCC and liver failure were selected to draw a network. The fact that there are just 54 journals in the largest journal cooperation network, however, suggests that there is a dearth of efficient journal-to-journal communication and interaction. To further advance the in-depth development of HCC and liver failure research, we should work to increase the scope of the journal cooperation network, fortify cooperation among journals, and encourage closer academic exchanges and information sharing in the future. Researchers can quickly access the latest research findings and cutting-edge developments within their fields through collaboration and communication via academic journals, facilitating interdisciplinary integration. This also promotes interaction among researchers from different countries and regions, thereby enhancing global scientific cooperation. We concentrate on journals with a publication volume of ≥ 9 to gain a clear understanding of the primary research orientations and noteworthy accomplishments in this discipline (**Figures 3A, B**; **Table 1**, SDC, **Table 2**). The journals with the highest publication frequency were *World Journal of Gastroenterology* (N=124), *Journal of Hepatology* (N=92), *Hepatology* (N=81), *Transplantation Proceedings* (N=79), and *Liver International* (N=77). *Journal Of Hepatology* has the highest total citation frequency (N = 11347). The core journals of hepatocellular carcinoma and liver failure research are the *World Journal Of Gastroenterology*, *Journal Of Hepatology*, and *Hepatology*, and they are leaders and role models in this field (**Figure 3C**). Currently, the research priorities in leading journals within the domain of HCC and liver failure encompass cancer-related risk factors (14, 15), the application prospects of imaging technology (16, 17), the construction of models to predict cancer progression (18, 19), advanced treatment methods (20–22), and the development of treatment strategies targeting specific biomarkers (23, 24). The journal “Cancers” has emerged as a prominent publication in recent years, evidenced by its substantial publication volume (**Figure 3D**). Through a comprehensive analysis of articles from these journals, we foresee that future research will likely emphasize the risk prediction of HCC (25, 26), the treatment of liver failure (27), and management strategies (28). These research directions not only deepen the understanding of HCC and liver failure but also facilitate the development of novel therapeutic approaches, ultimately aiming to achieve personalized treatment for patients suffering from liver failure post-liver cancer.

**Table 2:** Top 10 authors and top 10 co-cited authors.

Ranking	Authors	Documents	Institution and country	H-index	Co-cited authors	Count	Institution and country	H-index
1	Huang, Yi-Hsiang	27	Taipei Veterans General Hospital, Taiwan	27	Llovet, Jm	1162	Icahn School of Medicine at Mount Sinai, USA	162
2	Kudo, Masatoshi	25	Kindai University, Japan	110	Bruix, J	951	University of Barcelona, Spain	137
3	Pawlik, Timothy M.	23	Ohio State University, USA	91	Kudo, M	559	Kindai University, Japan	110
4	Hou, Ming-Chih	19	National Yang Ming University, Taiwan	53	Rahbari, Nn	412	Heidelberg University, Germany	58
5	Cescon, Matteo	18	University of Bologna, Italy	54	El-Serag, Hb	400	Baylor College of Medicine, USA	120
6	Huo, Teh-Ia	18	Taipei Veterans General Hospital, Taiwan	43	Mazzaferro, V	347	National Cancer Institute, Italy	92
7	Singal, Amit G.	18	UT Southwestern Medical Center, USA	56	Liaw, Yf	313	Chang Gung Memorial Hospital, Taiwan	99
8	Cucchetti, Alessandro	17	University of Bologna, Italy	42	Lencioni, R	290	University of Pisa, Italy	76
9	Lau, Wan Yee	17	The Chinese University of Hong Kong, Hong Kong	78	Younossi, Zm	278	Inova Fairfax Hospital, USA	119
10	Pirra, Antonio Daniele	17	University of Bologna, Italy	38	Makuuchi, M	274	The University of Tokyo, Japan	88

**Figure 4:**
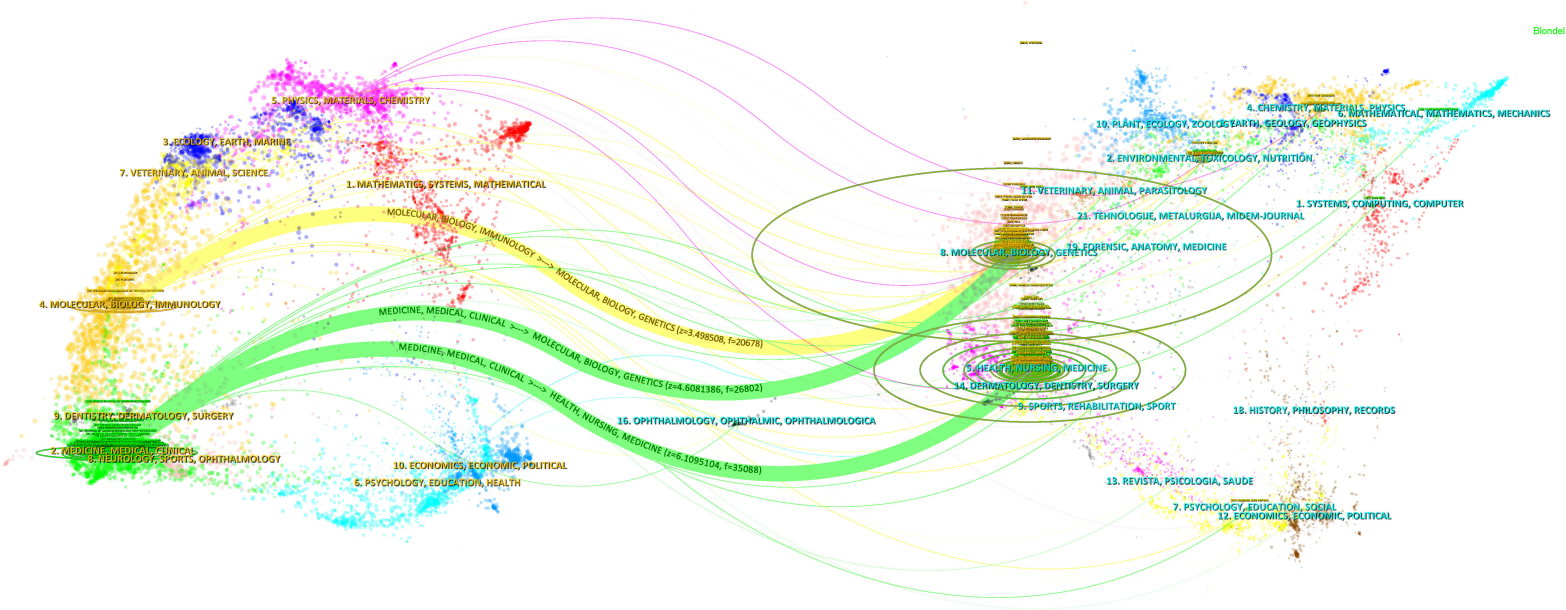
The journal dual overlay view. The citing journal is shown on the left, and the cited journal is on the right. The z-value represents the standardized citation count. Citation paths, colored in green and yellow, indicate the connections between the journals. The vertical axis of the ellipses represents the number of published papers, and the horizontal axis represents the number of authors.

The journal dual-map overlay created by CiteSpace provides a comprehensive and clear visualization of the sources of cited literature and the thematic distribution of journals (**Figure 4**). In the dual-map overlay of journals, the left side represents the citing journals, while the right side represents the cited journals. The curves indicate citation connections. The longer the vertical axis of an ellipse, the more papers the journal has published; the longer the horizontal axis, the more authors it has. The colored lines in the middle represent citation relationships. From the map, it can be seen that journals in molecular biology and immunology frequently cite journals in molecular biology, genetics, and biology (z = 3.498508, f = 20678); journals in medicine, medical, and clinical fields often cite journals in molecular biology, genetics, and biology, as well as journals in health, nursing, and medicine (z = 4.6081386, f = 26803; z = 6.01095, f = 35088).

### 3.3 Author and co-author analysis

**Figure 5:**
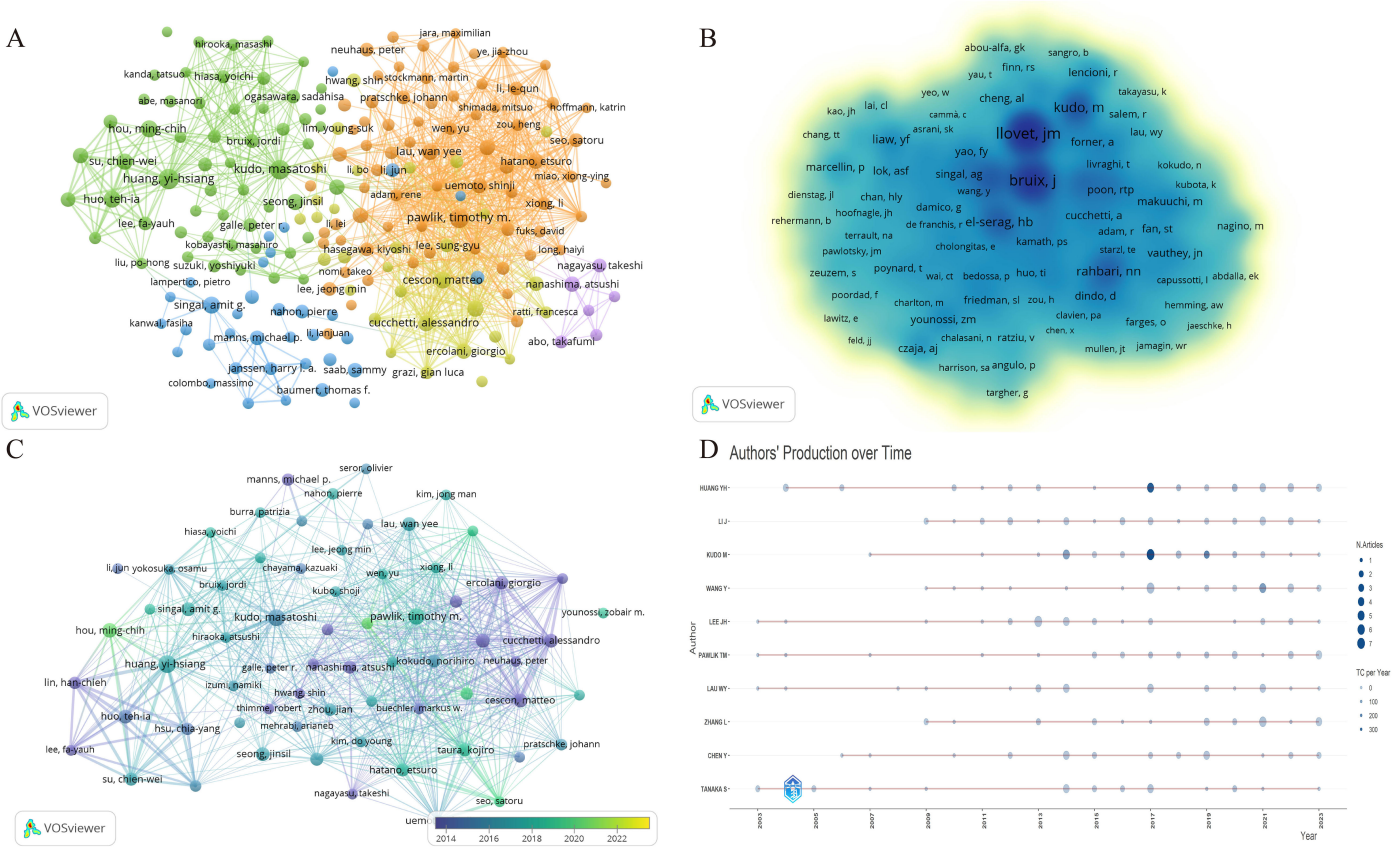
Visualization of the author network for hepatocellular carcinoma and liver failure. Each node represents an author or a co-authored author, with the size of the node indicating the number of publications, and the connections between the nodes representing collaborative relationships. **(A)** Collaborative network map of authors with ≥7 publications. **(B)** Core authors in hepatocellular carcinoma and liver failure. The nodes with darker colors represent greater importance in the field. **(C)** Emerging scholars in hepatocellular carcinoma and liver failure. The colors vary in accordance with the respective years. **(D)** Author’s production over time. Each line illustrates the annual publication and citation trends of each author. The node size represents the publication volume, while the color intensity indicates the citation count.

Our investigation revealed that a total of 23,228 authors have contributed to research in the domains of HCC and liver failure. Utilizing a criterion whereby only authors who have published seven or more papers were considered, we identified 200 significant contributors. These were subsequently categorized into five distinct author groups. The most populous of these groups comprised 72 researchers, signifying that collaborative research efforts in HCC and liver failure are still nascent (**Figure 5A**; **Table 2**). In **Figure 5A**, the largest network cluster features Cescon, and Matteo as pivotal figures, comprising 72 individuals and exhibiting a total link strength of 12,760. This highlights the critical role of Cescon, and Matteo in fostering global collaborative efforts.

Among the researchers, Huang Yi-Hsiang has published the highest number of papers, totaling 35, closely followed by Kudo Masatoshi with 27 publications, and others such as Pawlik Timothy (N =23), Hou Ming-Chih (N =19), Singal Amitg (N =18), Cescon Matteo (N =18), and Huo Tehia (N =18). Llovet JM stands out as the most cited author, with 1,162 citations, primarily focusing on the pathogenesis and treatment modalities of HCC (29, 30). Llovet JM and Bruix J are recognized as seminal figures in the study of HCC treatment and prognosis (31), as delineated in **Figure 5B**.

Emerging scholars such as Hou Ming-Chih and Li Lequn have also made notable contributions, recently publishing significant works (**Figure 5C**). Their team has identified the risk factors for recurrence prediction post-hepatectomy (32), including cirrhosis, surgical margin, and microvascular invasion. However, these findings necessitate further validation in conjunction with other clinical features to enhance the accuracy of prognostic assessments. Lastly, **Figure 5D** illustrates the temporal evolution of publication output among these authors.

### 3.4 Institutions

**Figure 6:**
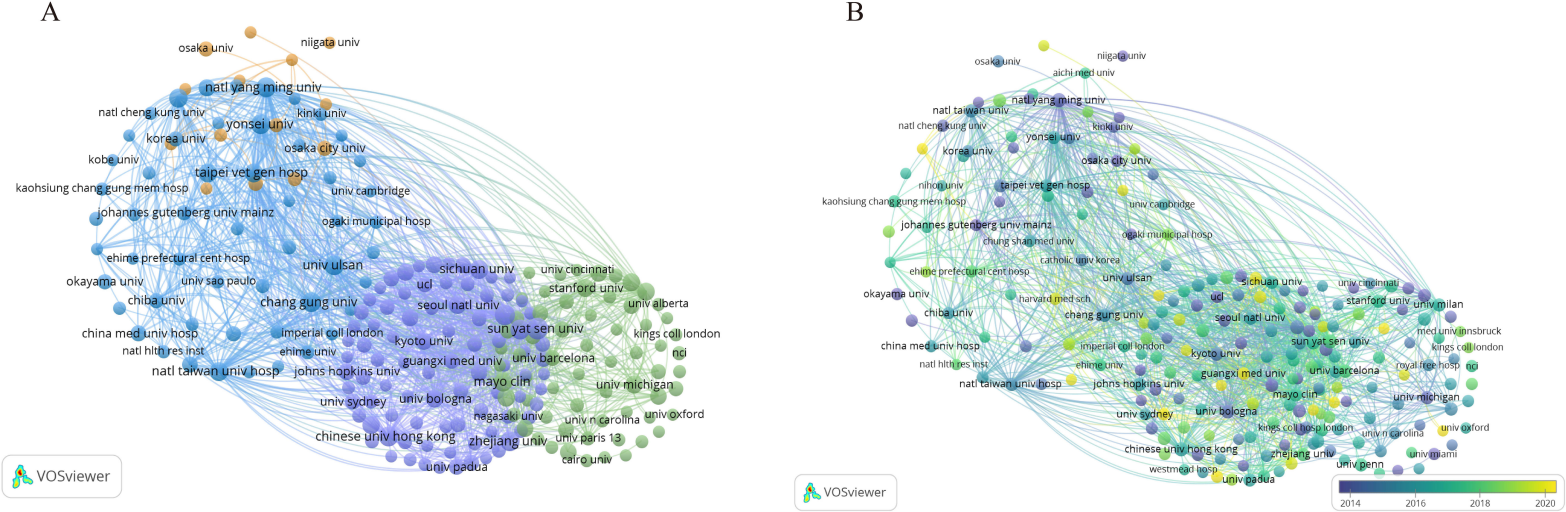
Visualization of Institutional Networks. Each node represents an institution, while the connections between nodes indicate collaborative relationships between institutions. **(A)** The institutional co-occurrence network map. **(B)** Emerging institutions in hepatocellular carcinoma and liver failure. The color represents a significant contribution to the field in the corresponding year.

To visually depict the collaboration between institutions and their research directions, we analyzed the publishing entities. The statistical results revealed that a cumulative total of 4,462 institutions were engaged in the scholarly discourse about HCC and liver failure. For visual analysis, we selected the top 219 institutions. The largest collaboration network consisted of 88 institutions and was divided into four main clusters (**Figure 6A**, **Table 3**, SDC, **Table 3**). This network is centered around Taipei Veterans General Hospital, which had 218 links and a total link strength of 53,282, followed by National Yang-Ming University and Sun Yat-sen University. Sun Yat-sen University published the most papers (N = 75), followed by the University of Hong Kong (N = 59). The highest citation frequency was observed for the Mayo Clinic (N = 6,024), followed by the University of Barcelona (N = 4,804) and the University of Hong Kong (N = 4,694). These institutions have established partnerships, participated in research projects, and supported and collaborated (**Figure 6B**). Additionally, current research in this field is primarily concentrated in universities. However, universities often focus on basic research and academic exploration, which may pose challenges in translating HCC and cirrhosis research findings into practical applications.

**Table 3:** Top 10 academic institutions in terms of publications.

Ranking	Institutions	Publications	Total link strength
1	Sun Yat-sen University	75	41
2	The University of Hong Kong	59	82
3	The Chinese University of Hong Kong	56	77
4	National Yang-Ming University	56	128
5	Zhejiang University	54	40
6	Fudan University	52	49
7	Chang Gung University	51	71
8	Yonsei University	51	66
9	University of Ulsan	50	63
10	Taipei Veterans General Hospital	49	136

### 3.5 Countries and regions

**Figure 7:**
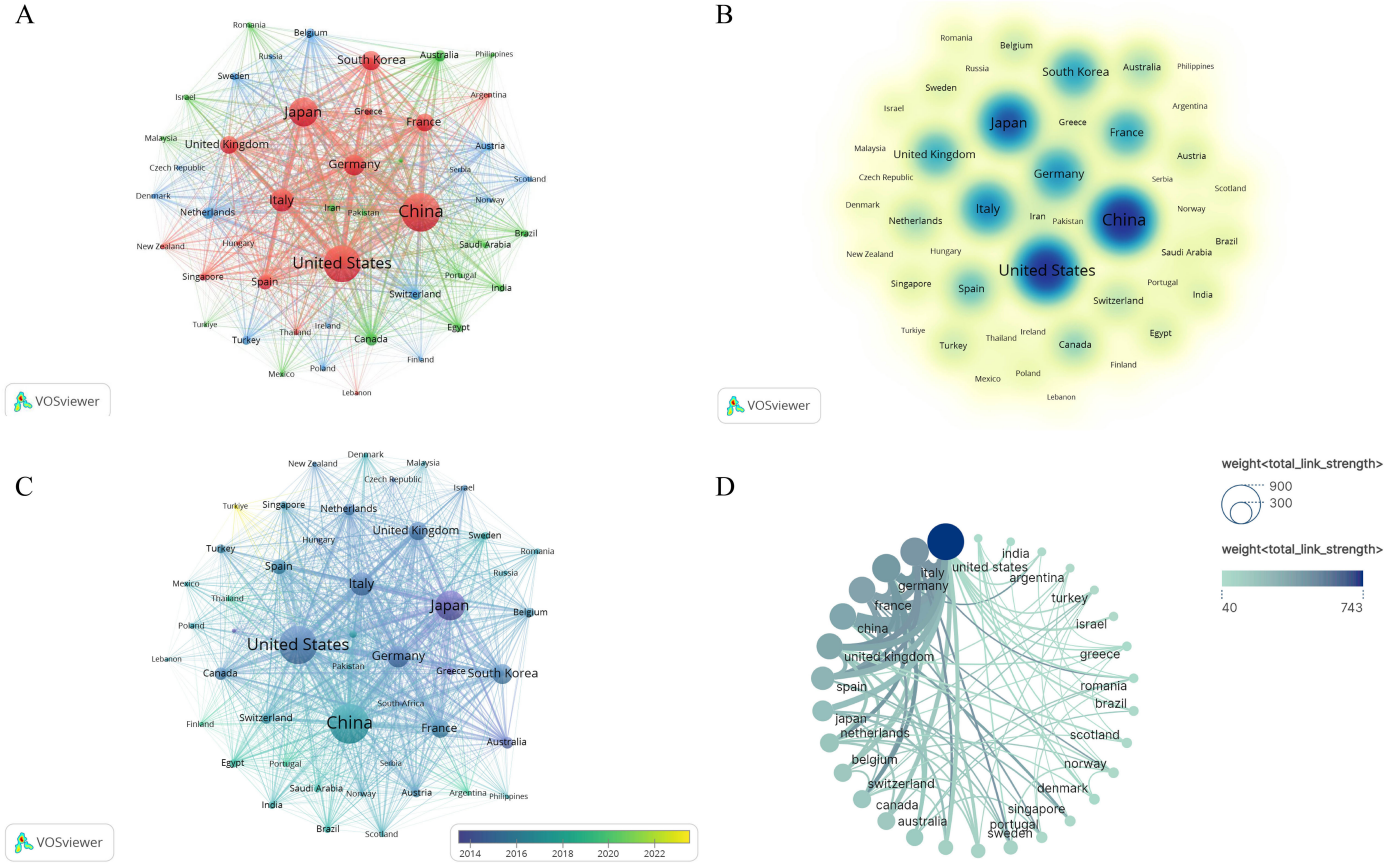
Collaborative Network Map of Counties-Regions. **(A)** Countries/Regions Cooperation Network Map. Each node represents a country, with the thickness of the connecting lines indicating the strength of the relationship. **(B)** Core Countries in hepatocellular carcinoma and liver failure. The darker the node color, the more significant the country. **(C)** Emerging Countries in hepatocellular carcinoma and liver failure. The color variations in the overlay view reflect the spatial mapping of the nodes based on the average publication year of each country. **(D)** Collaborative network map of countries. The size of each node corresponds to the volume of publications, and the thicker the line, the closer the collaboration between countries.

We utilized VOSviewer to analyze the publication data by country and region. To eliminate ambiguity in country names, we standardized them according to naming conventions, such as merging ‘England,’ ‘Scotland,’ and ‘Northern Ireland’ under the ‘United Kingdom.’ Researchers from 91 countries/regions contributed to studies on HCC and liver failure. We screened 49 countries with at least five publications, resulting in three major clusters (**Figure 7A**). The United States formed the most extensive national collaboration network, followed by China, Japan, and Italy. United States published the highest number of articles (N = 1002), followed by China (N =863) and Japan (N = 608). The United States had the highest number of citations (N = 64838), with Italy (N = 28163) and China (N = 24950) trailing behind. The United States, China, Italy, and Japan are central participants in this field (**Figure 7B**, **Table 4**), demonstrating significant influence and achievements. In recent years, countries like Turkey, Argentina, and the Philippines have increasingly focused on research in this domain (**Figure 7C**). **Figure 7D** is a map of the national cooperation network, and we can see that China has close cooperation with the United States, Italy, and Japan.

**Table 4:** Top 10 countries in terms of documents and citations.

Ranking	Country	Documents	Total link strength	Country	Citations
1	USA	1002	4995	USA	64838
2	China	863	4819	Italy	28163
3	Japan	608	4189	China	24950
4	Italy	382	3824	Germany	22102
5	Germany	324	2654	Japan	21808
6	South Korea	265	1416	France	21737
7	France	241	2896	England	18561
8	Taiwan	239	1387	Spain	16466
9	England	225	2199	Taiwan	11592
10	Spain	159	1967	Canada	9030

### 3.6 Keywords co-occurrence, clusters and bursts

**Figure 8:**
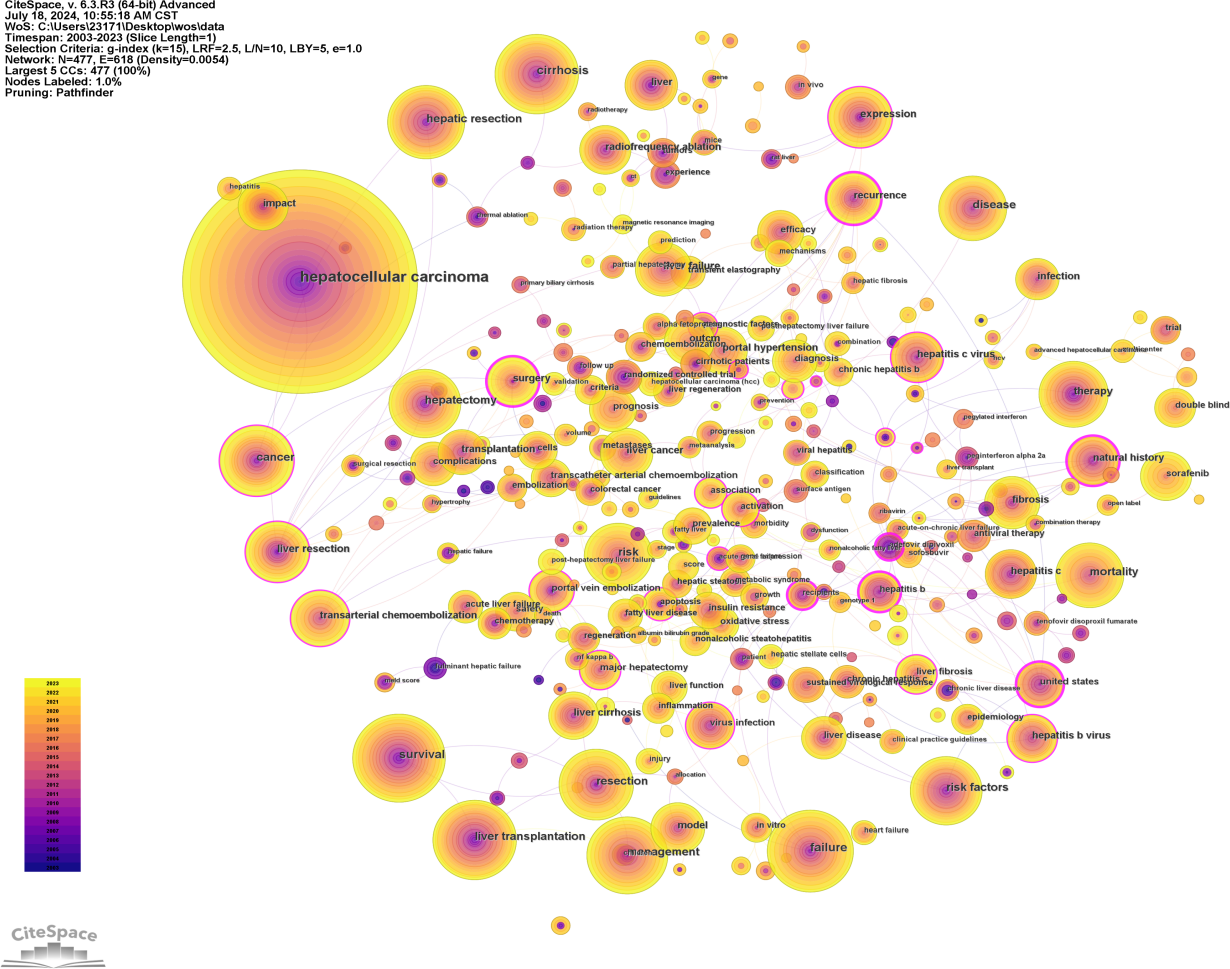
Network visualization of co-occurring keywords in hepatocellular carcinoma and liver failure. The nodes represent keywords, with the size indicating the frequency of their occurrence. The outer purple transparent circle represents the intermediary centrality of the node. A visible purple circle signifies that the keyword is highly important in the field. The connecting lines indicate that the keywords are cited within the same article.

Using CiteSpace software for keyword analysis, 447 keywords were identified in the literature (**Figure 8**, **Supplementary Table 4**). Aside from hepatocellular carcinoma and liver failure, the most frequent keywords included survival (N = 395), cirrhosis (N = 370), liver transplantation (N = 336), management (N = 310), hepatic resection (N = 308), and risk factors (N = 290). Keywords associated with the characteristics and development of liver failure in HCC encompassed cirrhosis (N = 370), liver transplantation (N = 336), and recurrence (N = 167). The most common treatments identified were liver transplantation (N = 336), hepatic resection (N = 308), hepatectomy (N = 256), liver resection (N = 203), and transarterial chemoembolization (N = 169). Keywords related to etiology included hepatitis B virus (N = 156), and hepatitis C virus (N = 155).

**Figure 9:**
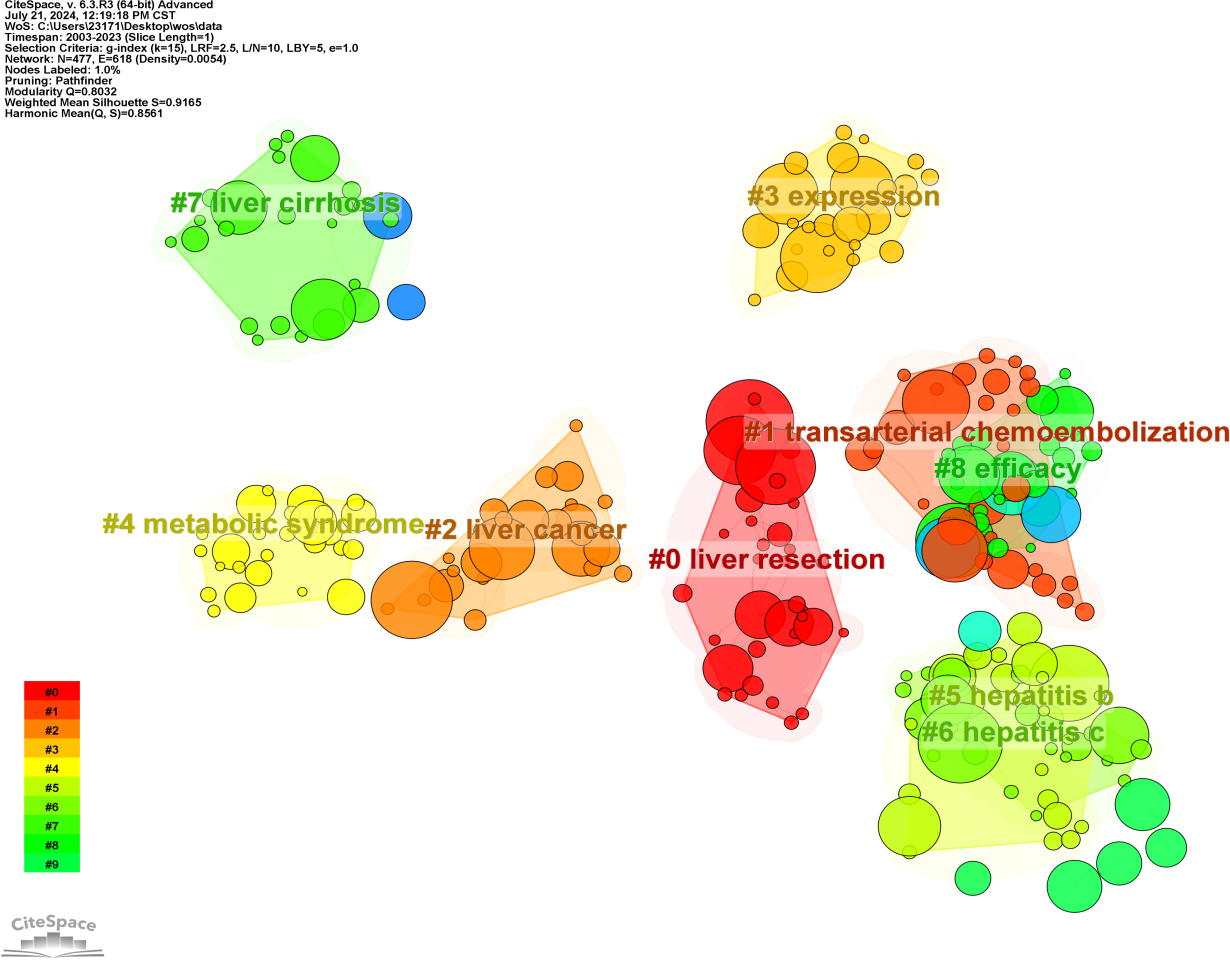
Clustering of Nine Key Terms in hepatocellular carcinoma and liver failure. Generate appropriate cluster titles based on the keywords within each cluster.

To elucidate the research hotspots within this domain, the log-likelihood ratio method was employed to cluster the keywords associated with HCC and liver failure based on their co-occurrence (**Figure 9**). The nine clusters with the highest research frequency were selected for detailed analysis. These clusters predominantly pertain to the etiology and treatment of HCC and liver failure. Specifically, the deep red clusters focus on first-line surgical treatments related to HCC and liver failure. Light red clusters are associated with radiotherapy and chemotherapy for HCC. Orange and dark yellow clusters are primarily related to HCC tumor markers and metastasis. Dark green and light green clusters are mainly associated with the etiology and complications of HCC and liver failure and the effectiveness of drug treatments. Yellow clusters focus on the classification and subtyping of HCC.

**Figure 10:**
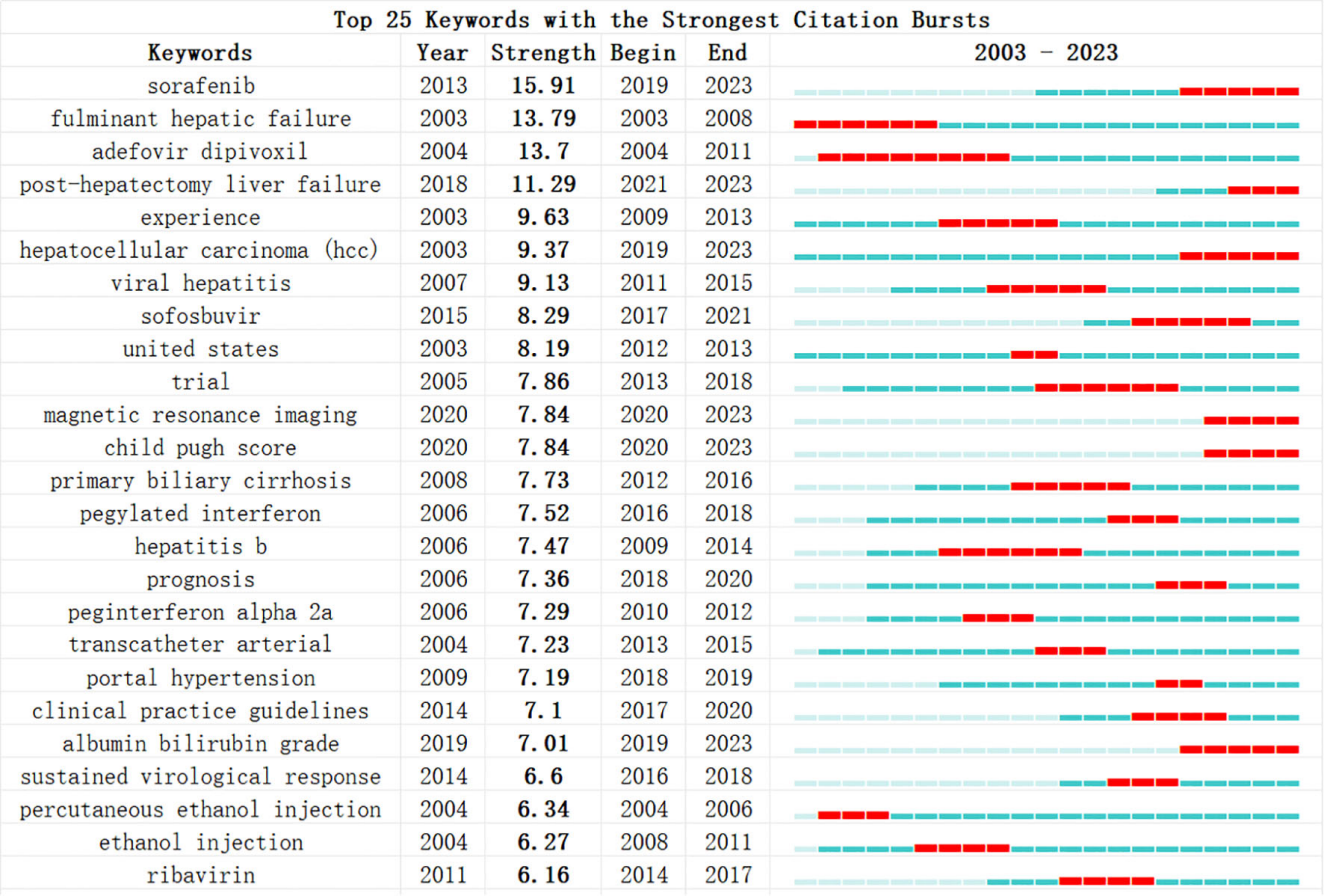
Most explosive keywords for hepatocellular carcinoma and liver failure. The blue bars show the time intervals, whereas the red bars highlight the burst periods. The beginning and ending years of these bursts are also provided.

**Figure 11:**
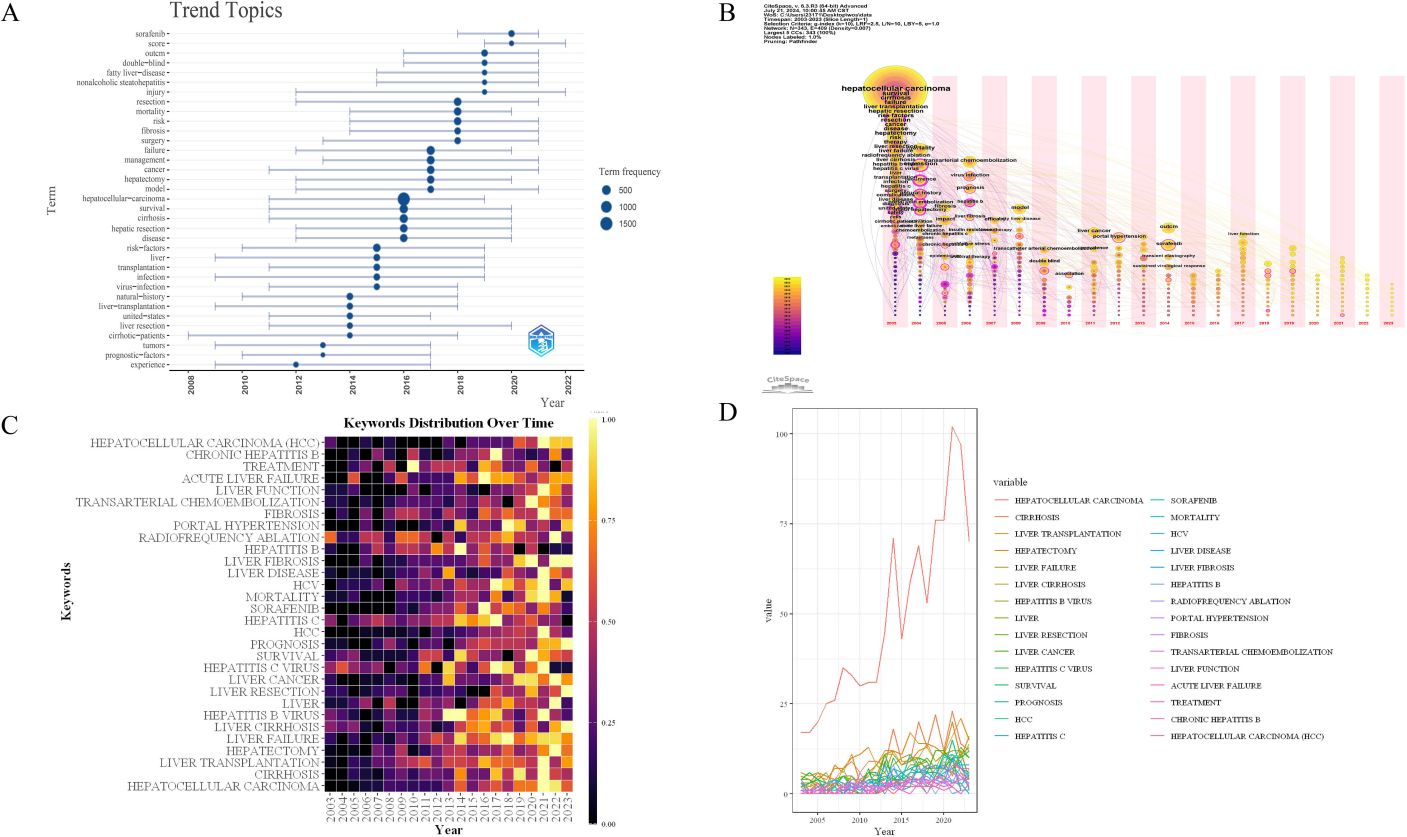
Timeline mapping of hepatocellular carcinoma and liver failure domain keywords. **(A)** keyword year evolution analysis. Node size represents the frequency of occurrence. **(B)** clustering timeline analysis. **(C)** keyword time hotspot graph. Each grid represents the popularity of keywords for the corresponding year, with lighter colors indicating a higher intensity of the surge, closer to the 1. **(D)** keyword timeline graph. Each line represents the evolution of a keyword in the past twenty years.

Additionally, we conducted a keyword burst analysis (**Figure 10**) and, a keyword year evolution analysis (**Figure 11**). **Figure 10** shows the top 25 keywords of the outbreak intensity. The most popular keywords in the past three years include sorafenib, post hepatectomy liver failure, liver function, nonalcoholic steatohepatitis, hepatic stellate cells, child-pugh score, etc. **Figure 11A** analyzes the keywords over two years, arranged from top to bottom based on their frequency during different periods. In **Figure 11B**, the node colors vary in intensity with time, with keywords from different years displayed from left to right. The connecting lines represent the strength of the relationships between the keywords. **Figure 11C** conveys the same meaning, with lighter colors indicating more popular keywords. **Figure 11D** presents the trends in keyword popularity over the past 20 years. The figures reveal that since 2003, keywords such as cirrhosis, liver transplantation, hepatectomy, and hepatitis B virus have garnered significant attention from scholars. Over the past three years, the primary research focus in the field of HCC and liver failure has transitioned from etiology and basic treatment to novel treatment options and patient prognosis. Current research predominantly concentrates on the drug sorafenib, liver cancer scoring systems, double-blind trials, and non-alcoholic and alcoholic fatty liver diseases. Moreover, there is a growing scholarly interest in the complications, disease progression, and treatment strategies for HCC and liver failure. Exploring new therapeutic drugs aims to reduce ineffective treatments and minimize unnecessary side effects and costs. These areas represent the critical directions for future research.

### 3.7 Co-cited reference analysis

**Figure 12:**
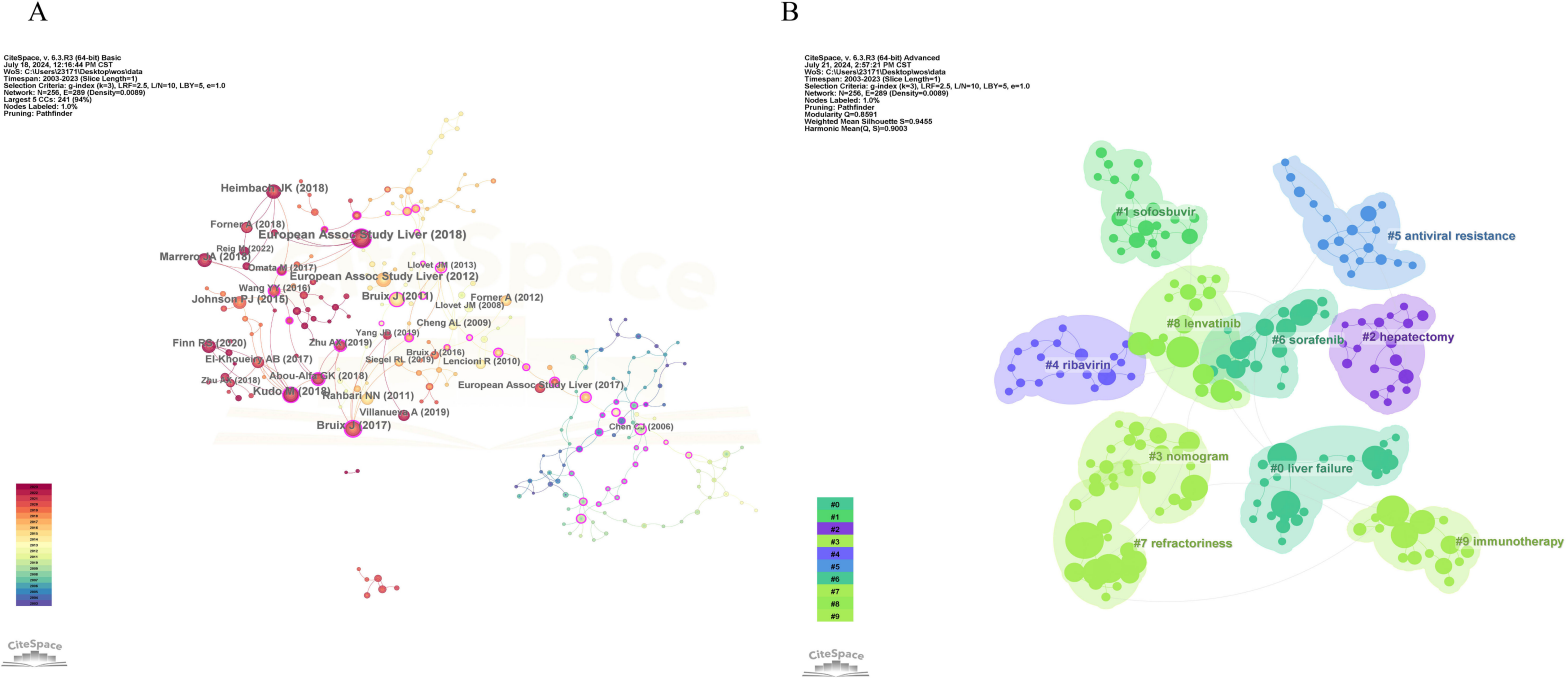
Co-citation visualization analysis of hepatocellular carcinoma and liver failure. **(A)** Visualization of citations. Each node represents an article, and the connection represents being cited together by the same article. **(B)** Visualization of literature clustering. The main clustering based on the content of the cited literature.

The co-citation analysis of literature is a crucial method for uncovering the interconnections between various works. Conducting a co-citation analysis of publications in the fields of HCC and liver failure can help us understand the forefront advancements, primary research directions, and highly influential documents in these areas. We employed CiteSpace software to perform co-citation and burst intensity analyses on the included literature (**Figure 12A**). The research on HCC and liver failure can be broadly divided into three phases: before 2011, studies mainly focused on the risk factors of HCC (33); from 2012 to 2017, attention shifted towards clinical trials and the exploration of new therapeutic methods and drugs for HCC (34, 35); since 2018, the focus has further deepened, emphasizing precision medicine and personalized treatment strategies (36). Researchers have increasingly concentrated on identifying new biomarkers and techniques to determine potential therapeutic targets and strategies.

We conducted clustering of the literature (**Figure 12B**) and identified ten major research areas in HCC and liver failure: Cluster 0 emphasizes complications and current detection methods, including stereotactic ablative radiotherapy and postoperative complications, aiming to enhance patient prognosis evaluation and personalized treatment. Cluster 1 mainly focuses on HCC drugs, including paritaprevir and ledipasvir, aiming to discover new drugs and provide more effective treatments. Cluster 2 addresses surgical treatments, including the indocyanine green clearance test and hepatectomy, aiming to improve surgical efficacy. Cluster 3 focuses on blood biomarkers, including albumin-bilirubin and nomogram, aiming to provide more sensitive markers. Cluster 4 assesses drug efficacy, including pegylated interferon and ribavirin. Cluster 5 concentrates on the study of drug resistance, including interferon and antiviral resistance, which aids in discovering new resistance genes or targets and enhances patient sensitivity to drug formulations. Cluster 6 focuses on molecular targeted therapy and efficacy assessment, including multikinase inhibitors and clinical trials, helping evaluate patient treatment responses and explore new biomarkers. Cluster 7 is related to mechanism studies, including surveys and deep learning. Cluster 8 is associated with biologic monoclonal antibodies, including regorafenib and nivolumab, significantly improving patient quality of life and prognosis with the application of biologics. Cluster 9 focuses on immunotherapy and metabolism, including metabolism and lenvatinib, providing new insights for disease treatment. Cluster 10 pertains to radiofrequency ablation, including radiofrequency ablation itself, where new technical methods have significantly increased patient survival rates.

**Figure 13:**
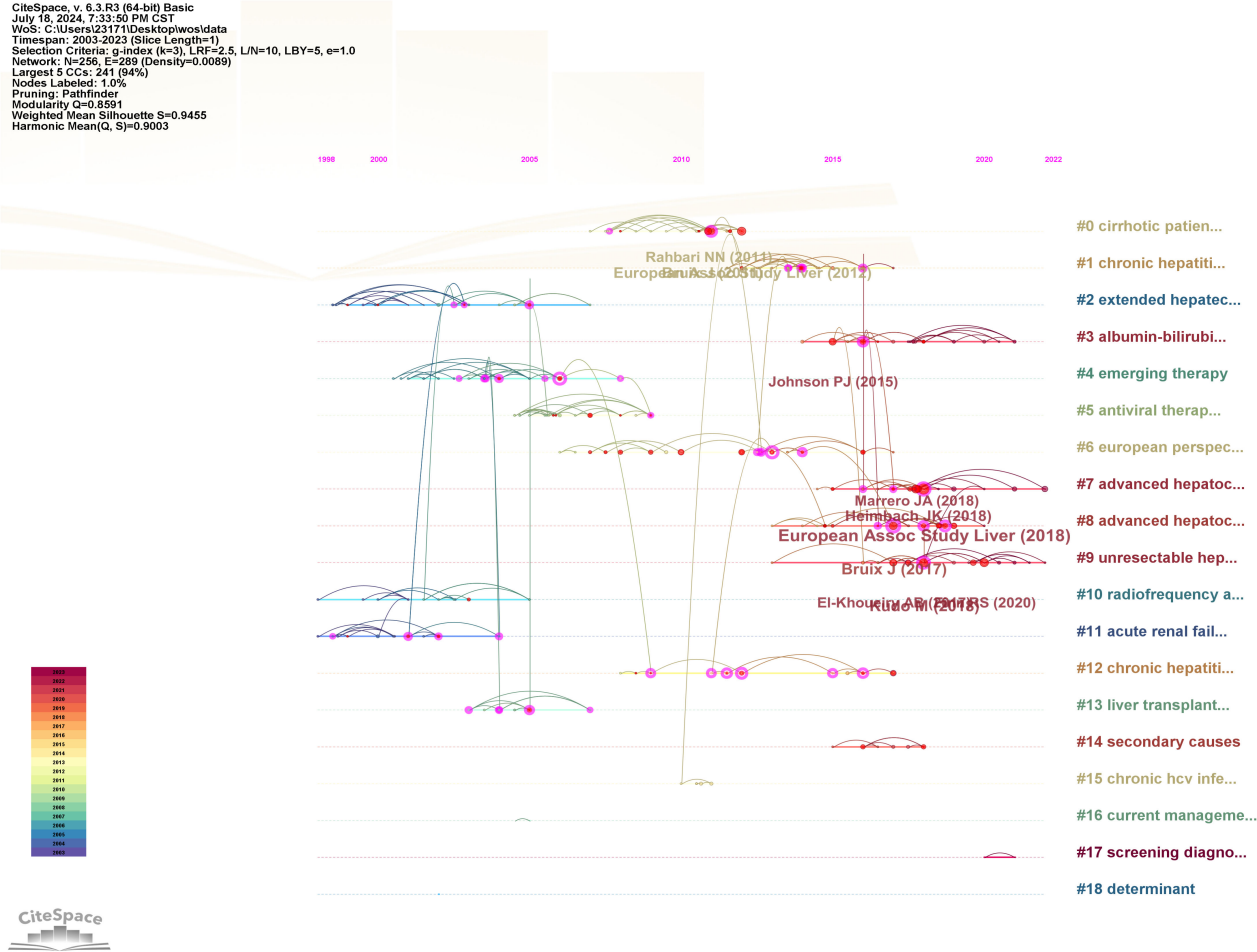
Evolution analysis of co-cited literature for hepatocellular carcinoma and liver failure. Each node represents a cited paper, with the connecting lines indicating co-citation with the same article.

We have listed the ten most relevant articles to the fields of HCC and liver failure in SDC, **Supplementary Table 5**, and the results of the analysis presented in **Figure 13** illustrate the evolution of co-cited literature, aiding in understanding the development of the field and identifying future research trends. The current research hotspots are mainly in targeted therapy, immunotherapy, and the exploration of new therapeutic approaches (37). It is foreseeable that immunotherapy-related literature may have higher citation frequencies in the future, and papers in this field may continue to receive significant attention.

### 3.8 Enrichment analysis

**Figure 14:**
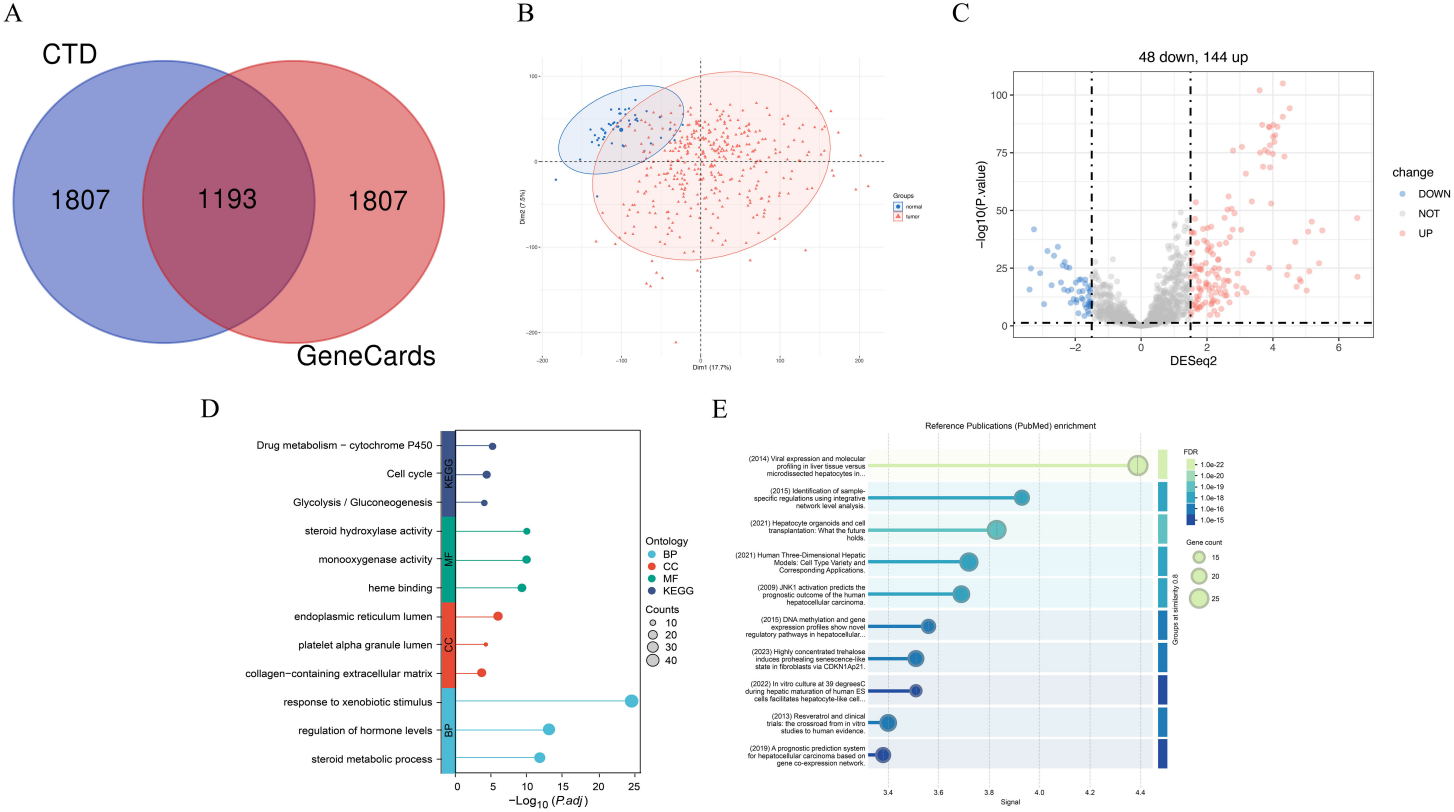
The enrichment analysis results of hepatocellular carcinoma and liver failure associated genes. **(A)** Overlap of common genes between liver failure-related genes from the CTD and GeneCards databases. **(B)** Principal component analysis. **(C)** Volcano plot illustrating differentially expressed genes. Up-regulated genes (n=144) are highlighted in red, while down-regulated genes (n=44) are shown in blue. **(D)** GO and KEGG enrichment analysis of the intersecting genes. **(E)** Enrichment analysis of differential gene-related PubMed database. FDR is a correction method for multiple hypothesis testing, which is used to control the proportion of false positive results. Signal usually refers to the correlation strength score between terms and target gene sets in the literature.

We identified 1,193 core genes associated with hepatic failure by intersecting data from the GeneCards and CTD databases (**Figure 14A**). Principal component analysis (PCA) (**Figure 14B**) demonstrated a clear separation between tumor and normal groups, indicating distinct expression patterns between the two conditions. Moreover, the sample distribution appeared uniform, with no evidence of clustering driven by non-biological factors, suggesting minimal batch effects. Differential expression analysis using the DESeq2 algorithm identified 192 differentially expressed genes among the core genes related to HCC and hepatic failure, comprising 144 upregulated and 48 downregulated genes (**Figure 14C**).

Gene ontology and pathway analyses (**Figure 14D**) revealed that these DEGs converge on critical biological processes implicated in liver dysfunction and malignant transformation. Enriched pathways, such as Drug metabolism − cytochrome P450 and response to xenobiotic stimulus, underscore the liver’s pivotal role in detoxification. Dysregulation of these pathways may contribute to carcinogen activation (e.g., via P450-mediated pro-carcinogen metabolism) and impaired toxin clearance in hepatic failure (38, 39). The enrichment of cell cycle-related pathways aligns with the unchecked proliferation characteristic of HCC, while glycolysis/gluconeogenesis pathway enrichment highlights the metabolic reprogramming commonly seen in cancer (40). At the molecular function level, enriched terms such as steroid hydroxylase activity and heme binding suggest disruptions in steroid hormone metabolism, which is linked to HCC progression, and oxidative stress responses, which exacerbate hepatocyte damage in both HCC and hepatic failure (41). Additionally, cellular component terms such as collagen-containing extracellular matrix and endoplasmic reticulum (ER) lumen point to fibrotic remodeling and ER stress-induced apoptosis, both of which play crucial roles in cirrhotic carcinogenesis and liver failure (42). Notably, enriched terms such as regulation of hormone levels and steroid metabolic processes may reflect the interplay between metabolic dysfunction in HCC (e.g., altered bile acid signaling) and systemic hormonal imbalances in hepatic failure (43). These overlapping molecular signatures emphasize how dysregulation of detoxification, proliferative signaling, and metabolic homeostasis contributes to both malignant transformation and functional liver decompensation, with cytochrome P450 activity emerging as a potential target for dual therapeutic intervention.

To further explore literature-based evidence linking the identified core genes with HCC and hepatic failure, we utilized STRING to generate a PubMed enrichment map (**Figure 14E**, **Supplementary Table 6**). This method ranks publications based on the association strength (signal score) between the documented biological terms and our selected genes, enabling the selection of highly relevant, well-supported studies for visualization. We prioritized the top ten publications for detailed representation. One study identified laser capture microdissection (LCM) as a crucial tool for validating HCC-associated gene signatures and identifying potential drivers of hepatocarcinogenesis, paving the way for novel diagnostic biomarkers and therapeutic targets (44). Another study highlighted the latest advancements in hepatic organoid models and their potential applications in regenerative medicine (45). Additionally, research on 3D human liver models demonstrated their ability to overcome limitations posed by traditional 2D cell culture systems and interspecies variability in drug metabolism enzymes and transporters, thereby improving disease modeling and drug development strategies (46). These clinically relevant, translational studies, closely associated with our identified core genes, provide broader insights into tackling HCC and hepatic failure and identifying novel therapeutic opportunities.

## 4 Discussion

### 4.1 General information

This study uses bibliometric methods to analyze the research progress on HCC and liver failure from 2003 to 2023. The analysis of the articles shows that the trend of increasing articles year by year reflects the improvement of research attention in this field. The top three countries in terms of publication volume produced 2,157 articles, accounting for 51.2% of the total output. Among the 47 countries analyzed, the United States, China, and Japan dominated the field. Moreover, China and the United States demonstrated the strongest international collaboration. These findings establish the crucial role and authoritative status of the United States and China in HCC and liver failure research. The United States, with its robust economy and highly active researchers, stands as the most influential country in this domain. China and Japan exhibit high productivity in this field, indicating significant attention to the disease. Extensive international collaboration in the future will greatly enhance the overall quality of research in this area.

Among the top 10 institutions ranked by the number of published papers, China has seven, while South Korea has three. The three institutions with the highest number of publications are all located in China. Sun Yat-sen University and the University of Hong Kong are the universities with the most published papers. They are the primary driving forces in HCC and liver failure research. Additionally, Taipei Veterans General Hospital and National Yang-Ming University maintain close ties and collaboration. This underscores the importance of seeking comprehensive partnerships among institutions.

Analyzing academic publications aids researchers in identifying suitable journals for submitting their articles within their fields. Peer-reviewed journals are crucial for academic publishing. The World Journal of Gastroenterology has the highest number of published papers, with 124 articles and an impact factor of 6.9. Overall, journals that frequently publish research on HCC and liver failure are primarily classified as Q1-Q2. Enhancing the global impact of these relevant journals is necessary.

### 4.2 Research basics and hot spots

The research hotspots can be analyzed from publications, references, keywords, etc. The frequently occurring keywords include targeted therapeutic drugs such as “Sorafenib”, “Adefovir dipivoxil”, “Sofosbuvir”, as well as terms related to the etiology of HCC and liver failure, such as “hepatitis B” and “nonalcoholic steatohepatitis”. Sorafenib, a multi-kinase inhibitor, has been a cornerstone of systemic therapy for advanced HCC (47). Clinical trials have shown that sorafenib significantly improves overall survival and delays disease progression in patients with advanced HCC. However, its benefits are often limited by adverse effects and the development of resistance, prompting the exploration of combination therapies and new drugs to enhance treatment outcomes (48). Currently, various combination therapies are available, with the FDA approving the combination of atezolizumab and bevacizumab for the treatment of liver cancer. In clinical trials, this combination therapy has demonstrated higher tumor response rates and longer progression-free survival than sorafenib (49, 50). Combination immunotherapy, such as the pairing of durvalumab and tremelimumab, is used for treating patients with unresectable advanced liver cancer. This combination has shown significant efficacy in clinical trials (51). Enzyme-activated anticancer drugs, where a specific enzyme SULT1A1 converts a class of compounds into anticancer agents, have demonstrated the ability to kill liver cancer cells in cell and mouse models, potentially representing a new direction for future liver cancer treatment (52). Alternatively, gene therapy introducing miR-22 (a naturally occurring small RNA molecule) can effectively inhibit the progression of liver cancer. Compared to existing drugs, miR-22 therapy shows longer survival times without toxic reactions (53). Additionally, drugs targeting the tumor microenvironment, such as oncolytic virus therapy, achieve therapeutic goals by using viruses to directly attack tumor cells (54). More new targets, new structures, and new types of biological agents provide additional options for the treatment of HCC and liver failure.

The most cited literature primarily consists of clinical practice guidelines providing the latest recommendations for clinical management (36, 55, 56) of HCC patients (57). In a phase III clinical trial comparing the efficacy of Lenvatinib and Sorafenib as first-line treatments for patients with unresectable HCC, it was found that among 11 patient deaths in the Lenvatinib treatment group, 3 were due to liver failure (48). Another clinical trial demonstrated that Regorafenib provided survival benefits for HCC patients with disease progression following Sorafenib treatment, with 2 deaths in the placebo group attributed to liver failure (58). A separate study reported that Atezolizumab combined with Bevacizumab achieved superior overall survival and progression-free survival compared to Sorafenib in patients with unresectable HCC. However, 38% of patients experienced severe toxicity, consistent with previous studies on these drugs (37). These findings highlight the critical importance of preventing liver failure as a key factor in improving patient outcomes. Another study introduced the albumin-bilirubin (ALBI) grade, which is commonly used in risk prediction and provides a simple, evidence-based, objective, and discriminatory method for assessing liver function in HCC, widely validated in international settings (59). In summary, many researchers focus on the exploration of new treatment methods and the effective monitoring and evaluation of liver function in this field. Based on the above research, we selected the following two hot topics to elaborate on in detail.

#### 4.2.1 Post-hepatectomy liver failure

PHLF is a severe complication following liver resection. Despite advances in surgical techniques and perioperative care, PHLF remains a critical issue. Recent studies have focused on elucidating the complex mechanisms underlying PHLF. Key findings suggest that oxidative stress, inflammatory response, and impaired liver regeneration are central to the pathogenesis of PHLF (60). Furthermore, the role of mitochondrial dysfunction and endoplasmic reticulum stress has been increasingly recognized (60). Advances in risk assessment tools and prediction models have been pivotal in identifying patients at high risk for PHLF (61). Incorporating biomarkers, such as serum liver function tests and imaging modalities like magnetic resonance imaging (MRI), into predictive algorithms has improved the accuracy of preoperative risk stratification (62, 63). Several studies have developed nomograms based on clinical assessments (Child-Pugh classification, liver stiffness, and portal hypertension) and the future liver remnant volume to predict symptomatic liver failure after liver resection for HCC (64–67). Elevated liver stiffness, with an optimal threshold of 9.5 Kilopascal (kPa), has been associated with the occurrence of symptomatic liver failure following HCC resection. Stratifying the safe limits of future liver remnant volume using these nomograms can assist surgeons in managing HCC resections (64). As mentioned above, ALBI serves as a significant prognostic factor for post-recurrence survival in HCC (68). ALBI score at the time of hepatectomy, as well as PHLF ≥ Grade B, were independently associated with ALBI score at recurrence (69). Spleen volume has also been strongly linked to PHLF (70). It has been identified as a predictor of PHLF and short-term mortality in HCC patients undergoing liver resection. The ratio of spleen volume to body surface area was significantly higher in the PHLF group, which also had a considerably poorer 1-year postoperative survival rate compared to the non-PHLF group (p = 0.044) (71). A single-center retrospective cohort study proposed that INR measurement on postoperative day 2 could be a predictor of PHLF (72). Enhanced recovery after surgery protocols and optimized perioperative care have significantly contributed to reducing PHLF incidence. With the continuous advancement of risk assessment tools and predictive models, particularly through the integration of biomarkers and imaging technologies, the accuracy of preoperative risk stratification is expected to improve, thereby optimizing the safety margins for liver resection and facilitating the development of personalized treatment and recovery pathways (73).

#### 4.2.2 Liver failure and drug-induced liver dysfunction

Drug-induced liver injury (DILI) is the main cause of acute liver failure (74). With the widespread adoption of targeted therapy and immunotherapy, DILI cannot be overlooked. Studies have found that anticancer drugs are the primary cause of DILI (75). Drugs causing DILI include antitumor drugs (26%), antibiotics (24%), analgesics (12%), and immune checkpoint inhibitors (75–78). In all cancer-targeted therapy drugs Multikinase inhibitors can cause liver damage, sometimes even irreversible, so early monitoring is essential (79). Nowadays, various models have been developed to monitor drug-induced liver injury (80). Liver magnetic resonance imaging omics models are used to predict DILI and γ-glutamyltranspeptidase-activated near-infrared fluorescent probes allow visualization of liver injury (81). Saliva metabolites are also promising non-invasive biomarkers for DILI (82). Protecting liver function during tumor treatment is extremely important for the good prognosis of patients. Some studies have shown that drugs such as tiopronin, magnesium isoglycyrrhizinate, and S-adenosylmethionine (AdoMet) may have potential hepatoprotective activity in the process of cancer treatment (83). However, well-designed prospective phase III randomized controlled trials are lacking to validate the necessity of introducing hepatoprotective agents in cancer patients, which may represent a future research direction (83).

Prognosis in HCC is closely tied to the ability to manage liver function while effectively treating the tumor. How to improve patient survival rates while protecting liver function, and minimize the occurrence of liver failure preoperatively, intraoperatively, and postoperatively, the development of novel anticancer drugs with lower liver toxicity and higher efficacy, as well as addressing risk factors related to liver failure such as hepatitis, are all critical issues that warrant further attention from researchers. Considering the early detection of tumor molecular characteristics and personalized medicine, as well as the integration of biomarkers, liquid biopsies, and patient-reported outcomes into clinical practice, there is potential to optimize treatment strategies and enhance our ability to predict and manage HCC and liver failure (84, 85).

### 4.3 Mechanical analysis

Liver cancer and liver failure, as two core issues in the spectrum of liver diseases, involve complex molecular networks and pathophysiological processes in their development. In recent years, keywords identified through bibliometric analysis, such as “sorafenib,” “viral hepatitis,” and “non-alcoholic steatohepatitis,” in conjunction with biological pathways like “IL-17 signaling pathway,” “response to xenobiotic stimulus,” “arachidonic acid metabolism,” and “steroid metabolic process,” as well as “cytochrome P450 metabolism,” have provided important clues for uncovering disease mechanisms. A systematic elucidation of the associations between these keywords and pathways can offer theoretical support for precision medicine.

Chronic viral hepatitis (such as HBV/HCV infection) is one of the main causes of liver cancer, with its pathological process closely linked to inflammation and immune disorders (86). The virus triggers the activation of hepatic stellate cells and promotes the formation of a fibrotic microenvironment by activating immune cells to release pro-inflammatory factors like IL-17 and TNF-α (87, 88). Tyrosine kinase inhibitors induce the inflammasome pathway, providing viable therapeutic targets for HCC (89). IL-17 promotes liver cancer cell migration and invasion by increasing the levels of IL-8, MMP2, and VEGF, while TNF-α exacerbates liver cell injury and apoptosis via the NF-κB pathway (90). TNF-α induces double-strand DNA breaks to increase hepatocyte apoptosis and exacerbates liver damage by enhancing the inflammatory response through the NF-κB pathway (91).

Regarding drug metabolism and hepatotoxicity, sorafenib, as a first-line targeted therapy for liver cancer, has its efficacy and toxicity heavily dependent on the metabolic activity of cytochrome P450 (CYP3A4) (92). CYP3A4 is primarily expressed in the liver and intestines and is involved in the metabolic activation and metabolism of various carcinogens (93). In liver failure patients, the reduced CYP450 system activity due to impaired liver function may lead to drug accumulation and exacerbate liver damage. Additionally, sorafenib affects angiogenesis by inhibiting the VEGFR/PDGFR pathway, which may indirectly worsen portal hypertension (94). To address this issue, pharmacokinetic studies are necessary to compare blood drug concentration differences between liver cancer and liver failure patients, combined with CYP3A4 genetic polymorphism analysis, to optimize individualized dosing regimens. Future studies could simulate the liver failure microenvironment to evaluate the effects of sorafenib on hepatocyte oxidative stress biomarkers, revealing the molecular basis of its hepatotoxicity. Sorafenib may influence the levels of epoxy metabolites derived from polyunsaturated fatty acids (PUFAs), as it is also a potent inhibitor of soluble epoxide hydrolase, which catalyzes the metabolism of PUFA-derived epoxy metabolites. In HCC patients treated with sorafenib, specific supplementation of omega-3 docosahexaenoic acid could increase the levels of the epoxy compound 19,20-EDP, which has potential anti-tumor activity (95).

Abnormal lipid metabolism plays a crucial role in the progression of non-alcoholic steatohepatitis (NASH) to liver cancer. Studies have found that chronic ethanol consumption enriches HBV-enhanced abnormal lipid metabolism through the HBx/SWELL1/arachidonic acid signaling pathway and activates Tregs (96). Arachidonic acid regulates inflammation through its metabolites such as leukotriene B4, thromboxane A2, or prostaglandin E2 (97). Prostaglandin E2 (PGE2), a metabolite of arachidonic acid, plays an indispensable role in liver health and disease and may be targeted for the development of liver-protective drugs targeting the COXs/PGESs/PGE2/EPs axis (98). Further investigation of the COX-2 gene may confirm its protective role in liver cancer induced by lipotoxicity, providing a basis for targeted interventions. Moreover, dysregulated lipoprotein metabolism exacerbates oxidative stress, driving DNA damage and carcinogenesis (99). Metabolomic analysis has shown that serum arachidonic acid metabolite levels are significantly elevated in NASH patients, suggesting their potential as early diagnostic markers (100). A high omega arachidonic acid/docosahexaenoic acid ratio may induce mitochondrial dysfunction and lipid metabolic changes in human liver cancer cells (101). Therefore, further studies on lipid metabolism will contribute to the prevention of liver cancer.

Future research can develop targeted drugs based on pathway mechanisms, such as IL-17 inhibitors and COX-2 antagonists, and assess their efficacy and safety through prospective clinical trials, with the potential for personalized treatment predictions. The molecular mechanisms of liver cancer and liver failure exhibit a multi-layered, multi-pathway interaction. By linking keywords with biological pathways, the core drivers of disease progression, such as inflammation, metabolic disorders, and oxidative stress, are revealed, providing innovative directions for interdisciplinary research. It is worth noting that the research results confirm that the hot spots of bioinformatics analysis and bibliometric analysis are consistent. Future efforts should integrate basic research with clinical practice to promote translational breakthroughs from mechanism analysis to therapeutic applications, ultimately improving patient prognosis.

### 4.4 Future trends

From the perspective of treatment techniques and drug development, immunotherapy and targeted therapies remain the key directions of growth. A bioactive nanomaterial, specifically exosomes derived from stem cells, has shown potential in treating acute liver failure induced by hepatectomy by modulating oxidative stress, reducing inflammation, and promoting hepatocyte regeneration (102). Moreover, kinase inhibitors have emerged as promising new tools for preventing liver failure (103). MicroRNA-based therapies hold significant potential for treating liver failure, although the development of novel delivery systems is still required (104). The remodeling of the tumor microenvironment is crucial for cancer treatment, with single-cell and spatial analyses revealing the co-localization of cancer stem cells and macrophages in hypoxic regions, which contribute to the poor prognosis of HCC (105). Electroacupuncture has been shown to activate the DMV cholinergic neuron-vagus nerve-macrophage axis in partial hepatectomy mice, promoting liver regeneration (106). Human hepatocyte organoids have demonstrated preclinical efficacy and safety in treating liver failure (107). Additionally, the development of various biomaterial scaffolds, such as fibrin gel scaffolds with adipose-derived stem cells, has been shown to enhance liver regeneration post-hepatectomy (108). Exosome-encapsulated oxidized hyaluronic acid hydrogels have also shown promise in promoting liver regeneration and post-hepatectomy applications (109). The continuous development of emerging technologies and drugs is expected to further advance the field.

In the realm of risk prediction and early diagnosis, medical imaging analysis, risk prediction model construction, and treatment response prediction using intelligent algorithms and machine learning have shown immense potential. Deep learning-based radiomics models have been applied in preoperative liver reserve function assessment and DILI prediction (110–114). The use of advanced imaging techniques to assess and predict post-hepatectomy liver failure is another key area of focus (62). Research has demonstrated that functional liver imaging scores can predict adverse events in HCC patients (115). Future studies could integrate multi-omics data to develop predictive systems that combine clinical features, radionics, and liquid biopsy for optimizing individualized surgical plans. These models will likely be further refined to incorporate additional biomarkers and imaging metrics, enhancing their predictive accuracy. The rapid development of DILI dynamic monitoring technologies is also noteworthy, with strategies such as SERS being used to detect mitochondrial autophagy in liver injury models induced by drugs like cisplatin (116). Fluorescent probes have enabled real-time *in vivo* imaging of DILI-related inflammatory responses (117), autophagy processes (118), lipid droplets, peroxides (119), carbon monoxide (120), and peroxynitrite (121).

In clinical practice, the Multidisciplinary Team model will continue to strengthen, promoting communication and collaboration between different specialties, thereby improving treatment outcomes and patient survival rates. HCC and liver failure are global health challenges that require coordinated efforts across nations. In the future, research institutions and healthcare organizations worldwide will strengthen collaboration, share research findings and clinical experiences, and jointly conduct large-scale clinical trials to accelerate the development and application of new drugs and technologies.

The integration of immunotherapy, nanotechnology, and predictive models represents a transformative frontier in HCC and liver failure management, poised to redefine precision medicine and bridge critical gaps between translational research and clinical implementation.

## 5 Limitations

Our study has several limitations. Firstly, we relied on the WOSCC database and included only articles and reviews published in English due to the limitations of visual software analysis, which might have resulted in some data omissions. Secondly, our interpretations of the number of publications, citation counts, and keywords may be somewhat subjective, leading to potential variability in conclusions among different researchers. Lastly, we cannot guarantee that every publication obtained fully adheres to the relevance criteria of our search. Moreover, the inclusion of literature in the past two decades may lead to the omission of the latest literature. The bioinformatics analysis relied solely on the TCGA-LIHC database, which restricts the generalizability of findings due to the lack of cross-validation with multi-omics datasets or diverse population cohorts. Despite these limitations, our analysis provides a comprehensive overview of the current state of the field.

## 6 Conclusion

This bibliometric analysis provides the first comprehensive mapping of the evolving synergy between HCC and liver failure research over two decades. Key findings reveal a paradigm shift from risk factor exploration to therapeutic innovation and personalized medicine, driven by advancements in immunotherapy, targeted therapies, and precision biomarkers. The United States and China dominate research output, while institutions like Sun Yat-sen University and Mayo Clinic lead in translational impact. Emerging hotspots include PHLF prediction models, DILI monitoring, and non-alcoholic steatohepatitis-related HCC. Enrichment analysis suggested that drug metabolism, cell cycle, lipid metabolism, oxidative stress and other signaling pathways play a central role in the disease.

Clinically, this study underscores the urgency of integrating radiomics, liquid biopsies, and multidisciplinary strategies to optimize risk stratification and therapeutic outcomes. Future priorities should focus on :(1) validating novel biomarkers (e.g., imaging-based liver stiffness, saliva metabolites) for early detection ;(2) refining PHLF prediction through machine learning and multi-omics integration ;(3) developing low-toxicity therapies (e.g., stem cell-derived exosomes, miR-22-based regimens); and (4) fostering global collaboration to address disparities in HCC management. These directions promise to bridge gaps between basic research and clinical practice, ultimately improving survival and quality of life for patients worldwide.

## Data Availability

The original contributions presented in the study are included in the article/**Supplementary Material**. Further inquiries can be directed to the corresponding authors.
