# Supplementary material for: Mapping the knowledge domains of literature on hepatocellular carcinoma and liver failure: a bibliometric approach
Source: Front Oncol. 2025 Apr 16;15:1529297. doi: 10.3389/fonc.2025.1529297 (PMC12040667; doi:10.3389/fonc.2025.1529297)
Supplement: Supplementary file 1 [file Table1.pdf]

Mapping the knowledge domains of literature on hepatocellular carcinoma and liver failure: A bibliometric approach

Supplemental Content

Supplementary Table 1. Terms and search strategy.

Terms	Entry Terms	Search strategy
Hepatocellular Carcinoma	Carcinoma, Hepatocellular；Cancer, Adult Liver；Carcinomas, Hepatocellular；Cancers, Adult Liver; Hepatocellular Carcinomas Liver Cancers, Adult; Hepatocellular Carcinoma; Liver Cell Carcinoma; Hepatoma; Carcinoma, Liver Cell; Hepatomas; Carcinomas, Liver Cell; Liver Cancer, Adult; Cell Carcinoma, Liver; Adult Liver Cancer; Cell Carcinomas, Liver; Adult Liver Cancers; Liver Cell Carcinomas; Liver Cell Carcinoma, Adult	(TS=("Carcinoma, Hepatocellular") OR TS=("Carcinomas, Hepatocellular") OR TS=("Hepatocellular Carcinomas") OR TS=("Hepatocellular Carcinoma") OR TS=("Hepatoma") OR TS=("Hepatomas") OR TS=("Liver Cancer, Adult") OR TS=("Adult Liver Cancer") OR TS=("Adult Liver Cancers") OR TS=("Cancer, Adult Liver") OR TS=("Cancers, Adult Liver") OR TS=("Liver Cancers, Adult") OR TS=("Liver Cell Carcinoma") OR TS=("Carcinoma, Liver Cell") OR TS=("Carcinomas, Liver Cell") OR TS=("Cell Carcinoma, Liver") OR TS=("Cell Carcinomas, Liver") OR TS=("Liver Cell Carcinomas") OR TS=("Liver Cell Carcinoma, Adult"))AND (TS=("Hepatic Failure") OR TS=("liver failure"))
Liver failure	Hepatic Failure	

Supplementary Table 2. Top 10 journals in terms of publications.

Ranking	Journal	Record Count	Citations	IF	H-index	Country	JCR
1	World Journal of Gastroenterology	124	4183	4.3	129	China	Q2
2	Journal Of Hepatology	92	11347	26.8	216	Netherlands	Q1
3	Hepatology	81	9829	12.9	326	United States	Q1
4	Transplantation Proceedings	79	862	0.8	77	United States	Q4
5	Liver International	77	2808	6	98	Denmark	Q2
6	Hepato-Gastroenterology	67	646	N/A	71	Switzerland	N/A
7	Plos One	64	1294	2.9	268	United States	Q2
8	Hepatology Research	60	1385	3.9	66	Japan	Q2
9	Liver Transplantation	60	3739	4.7	137	United States	Q1
10	Cancers	55	822	4.5	53	Switzerland	Q1

Supplementary Table 3. Top 10 academic institutions in terms of citations.

Ranking	Institutions	citations	Total link strength
1	Mayo Clinic	6024	63
2	University of Barcelona	4804	60
3	The University of Hong Kong	4694	82
4	University of Toronto	4061	44
5	University of Pittsburgh	3645	41
6	Icahn School of Medicine at Mount Sinai	3341	32
7	Hannover Medical School	3283	47
8	University of Michigan	3197	56
9	University of California, Los Angeles (UCLA)	3162	31
10	University of Milan (Università degli Studi di Milano)	3124	64

Supplementary Table 4. Top 10 keywords in frequency.

Ranking	Keywords	Count	Ranking	Keywords	Count
1	hepatocellular carcinoma	2375	6	management	310
2	survival	395	7	hepatic resection	308
3	cirrhosis	370	8	risk factors	290
4	failure	369	9	resection	285
5	liver transplantation	336	10	cancer	284

Supplementary Table 5. The top 10 co-cited articles related to HCC and liver failure.

Co-cited articles	Year	Count	Centrality
European Assoc Study Liver, 2018, J HEPATOL, V69, P182, DOI 10.1016/j.jhep.2018.03.019	2018	139	0.14
Kudo M, 2018, LANCET, V391, P1163, DOI 10.1016/S0140-6736(18)30207-1	2018	99	0.25
Bruix J, 2017, LANCET, V389, P56, DOI 10.1016/S0140-6736(16)32453-9	2017	96	0.48
Heimbach JK, 2018, HEPATOLOGY, V67, P358, DOI 10.1002/hep.29086	2018	87	0.04
European Assoc Study Liver, 2012, EUR J CANCER, V48, P599, DOI 10.1016/j.ejca.2011.12.021	2012	80	0.08
Bruix J, 2011, HEPATOLOGY, V53, P1020, DOI 10.1002/hep.24199	2011	79	0.28
Marrero JA, 2018, HEPATOLOGY, V68, P723, DOI 10.1002/hep.29913	2018	72	0.05
Johnson PJ, 2015, J CLIN ONCOL, V33, P550, DOI 10.1200/JCO.2014.57.9151	2015	71	0.05
Finn RS, 2020, NEW ENGL J MED, V382, P1894, DOI 10.1056/NEJMoa1915745	2020	68	0.06

Supplementary Table 6. Pubmed literature based on enrichment of 192 genes related to HCC and liver failure.

Publication	IF	JCR	Year	Count in network	Strength	Signal	False discovery rate
PMID:25141867	6.1	Q1	2014	26 of 83	1.48	4.39	7.14E-22
PMID:25928379	3.4	Q2	2015	19 of 43	1.63	3.93	1.82E-17
PMID:34663941	9.5	Q1	2021	24 of 86	1.43	3.83	4.99E-19
PMID:34631680	4.3	Q1	2021	23 of 82	1.43	3.72	2.52E-18
PMID:19686584	27.7	Q1	2009	21 of 66	1.49	3.69	1.87E-17
PMID:25945129	4.8	Q1	2015	18 of 46	1.58	3.56	5.71E-16
PMID:36609486	5.2	Q1	2023	20 of 63	1.49	3.51	1.93E-16
PMID:35338220	3.8	Q1	2022	16 of 33	1.67	3.51	3.79E-15
PMID:23448440	2.6	Q2	2013	21 of 76	1.43	3.4	1.93E-16
PMID:31086582	2.4	Q3	2019	19 of 60	1.49	3.38	9.72E-16
PMID:34238253	3.4	Q2	2021	14 of 23	1.77	3.38	3.96E-14
PMID:35047893	2.7	Q3	2020	18 of 53	1.52	3.34	2.7E-15
